# Sn–Fe Dual-Metallic Nanoparticles on S,N-Codoped g‑C_3_N_4_‑Derived Tubular Carbon as an Efficient Bifunctional Catalyst for Oxygen Reduction Reaction and Oxygen Evolution Reaction

**DOI:** 10.1021/acsami.6c00103

**Published:** 2026-04-25

**Authors:** Berhanu Telay Mekonnen, Daniel Manaye Kabtamu, Sun-Tang Chang, Guan-Cheng Chen, Amil Aligayev, Francisco Javier Dominguez-Gutierrez, Yao-Ming Wang, Sheng-Yu Wang, Wenyi Huo, Chen-Hao Wang

**Affiliations:** † Department of Materials Science and Engineering, 34878 National Taiwan University of Science and Technology, Taipei 106335, Taiwan; ‡ NOMATEN Centre of Excellence, 69701 National Centre for Nuclear Research, Otwock 05-400, Poland; § Maritime Innovation & Industry Promotion Department, Metal Industries Research & Development Centre, Kaohsiung 811160, Taiwan; ∥ Advanced Manufacturing Research Center, National Taiwan University of Science and Technology, Taipei 106335, Taiwan; ⊥ Research Center for Critical Issues, Academia Sinica, Tainan 711010, Taiwan

**Keywords:** oxygen reduction reaction, oxygen evolution reaction, anion exchange membrane fuel cell, bifunctional electrocatalyst, nonprecious metal catalyst

## Abstract

Developing cost-effective, high-performance bifunctional electrocatalysts for the oxygen reduction reaction (ORR) and oxygen evolution reaction (OER) is crucial for advanced clean energy technologies. This work details Sn–Fe bimetallic nanoparticles anchored on S,N-codoped graphitic carbon nitride (C_3_N_4_)-derived tubular carbon (SnFe/SNC_T), which are synthesized via a facile pyrolysis method at 850 °C (SnFe/SNC_850). The optimized SnFe/SNC_850 catalyst, which is characterized by a distinct bamboo-like tubular morphology, demonstrates superior ORR activity with a half-wave potential (*E*_1/2_) of 0.86 V vs RHE in 0.1 M KOH, surpassing commercial Pt/C (0.82 V). Furthermore, it exhibits excellent OER performance, requiring only 340 mV overpotential to achieve 10 mA cm^–2^, and displays remarkable overall bifunctionality. When SnFe/SNC_850 is integrated into an anion exchange membrane fuel cell (AEMFC), it delivers a peak power density of 277 mW cm^–2^, significantly outperforming Pt/C-based cells (168 mW cm^–2^). The catalyst also demonstrates exceptional durability, with only 20 mV of *E*_1/2_ decay after 30,000 cycles, compared to 50 mV for Pt/C. This enhanced performance is attributed to the synergistic interplay between Fe–N_*x*_/Fe–S_*x*_ active sites and intermetallic Fe_3_SnC/FeS domains. These findings establish SnFe/SNC_850 as a highly promising nonprecious-metal bifunctional electrocatalyst for practical energy-conversion applications, paving the way for sustainable clean-energy solutions.

## Graphical abstract

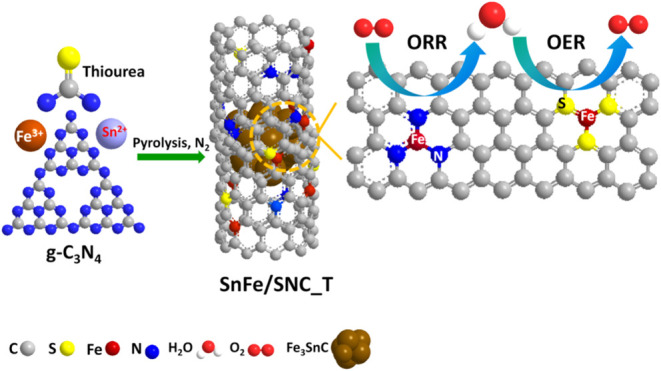

## 1 Introduction

The escalating global energy crisis and pressing environmental concerns have spurred intensive research into eco-friendly renewable energy technologies, among which fuel cells are considered highly promising for applications ranging from electric vehicles to portable electronic devices. However, the widespread commercialization of fuel cells is significantly hampered by several factors, most notably sluggish cathode oxygen reduction reaction (ORR) kinetics and reliance on expensive, scarce noble-metal catalysts, such as platinum (Pt).^1,2^ The ORR’s slow reaction rate inherently limits fuel cell performance and efficiency.

While Pt-based materials have traditionally served as benchmark ORR electrocatalysts, their prohibitive cost, limited reserves, and susceptibility to deactivation (e.g., CO poisoning and time-dependent performance degradation) severely restrict their large-scale deployment. Furthermore, efficient bifunctional electrocatalysts that can effectively drive the ORR and the oxygen evolution reaction (OER) are highly desirable for advanced energy systems like rechargeable metal-air batteries and regenerative fuel cells. The OER, the anodic counterpart to ORR, also suffers from slow kinetics and typically requires high overpotentials, underscoring the need for robust, cost-effective alternatives.

Consequently, extensive research efforts have been directed toward developing nonprecious metal catalysts (NPMCs) as viable alternatives. Among various NPMCs, transition-metal- and nitrogen-codoped carbon materials (M–N-C) have emerged as promising candidates for ORR and OER due to their potentially high activity, good durability, and low cost.^1,3–7^ However, achieving highly efficient bifunctional M–N-C catalysts that excel in ORR and OER remains a significant challenge, often requiring intricate material design and synthesis strategies. The synergistic coupling between multiple metallic components and the tailored nanostructure of the carbon support is considered crucial for optimizing bifunctional performance.^8–11^ For instance, incorporating bimetallic systems can offer richer redox chemistry and more active sites than single-metal counterparts. Moreover, heteroatom doping (e.g., S and N) into the carbon matrix can modulate electronic properties and create defect sites, thereby further enhancing catalytic activity. Similarly, a well-defined porous or hierarchical carbon architecture, such as tubular structures, can facilitate mass transport and expose more active sites.^12^ Composite electrocatalysts such as CoSn@NC,^13^ FeCo-SNC,^4^ Ni3FeN/VN-NG,^14^ Ni–Co-CPO-27,^15^ and CoFe/Se@CN^16^ have been reported as catalysts for ORR in fuel cells and rechargeable Zn-air batteries. Despite these achievements, the most widely investigated metal compounds focus mainly on 3d transition elements such as Ni, Co, Fe, Mn, Cu, and Zn. However, the intrinsic limitations of 3d transition metals, including their moderate catalytic activity and stability due to incomplete d-orbital filling, have spurred the exploration of alternative strategies.

On the other hand, owing to the partially occupied valence p-orbitals, main-group elements such as Sb, Sn, In, and Bi based electrocatalysts are attracting increasing research interest for N_2_ fixation and CO_2_ reduction in terms of single atomic sites^17–19^ or alloying with a second metal.^20,21^ This remarkable progress demonstrates the great potential of these materials in electrochemical applications. In this regard, Li et al. reported the bimetallic CoSn@NC for ORR in microbial fuel cells.^13^ This catalyst demonstrated superior bifunctional activity, accelerating ORR and improving antibacterial activity. The main group element Sn modulated the electronic structure of bimetallic CoSn by drawing electrons from the transition metal Co. This electron redistribution in CoSn@NC optimized the O_2_ adsorption at Co sites for rapid ORR kinetics. However, intensive studies are still lacking to fully explore the intrinsic catalytic potential of combining main-group and transition-metal elements for ORR.

Significant efforts have also been made to dual- and triple-heteroatom-doped M–N-Cs to improve further ORR performance.^4,22,23^ Dual or triple heteroatom doping alters the spin-charge densities and redistributes charge within the carbon matrix, thereby enhancing ORR activity.^4^ The N doping, resulting in C–N structures, such as pyridinic-N and graphitic-N, improves electron transfer. In contrast, S doping increases the positive charge and electronic spin density of carbon atoms, thereby enhancing oxygen adsorption.^4,5^ Besides, most encapsulated metal ions are reduced to metals or alloys at high temperatures, facilitating the formation of pyridinic C–N and graphitic C–N structures.^4,5^ Other metal atoms chelate with nitrogen atoms to form M-N_*x*_ or M-S_*x*_ moieties, which are widely recognized as active sites for ORR.^24,25^ Therefore, this strategy enhances ORR by *in situ* transforming metal atoms into metal nitrides or sulfides. This approach naturally integrates active ORR sites, providing superior bifunctional catalytic performance.

The g-C_3_N_4_ is a promising precursor for fabricating M-NC catalysts.^26–29^ It has a high nitrogen concentration, with an atomic ratio exceeding 57%, and the pyridine-like nitrogen’s lone-pair electrons are prone to adsorbing metal ions onto the g-C_3_N_4_ sheet.^26–29^ Furthermore, thiourea is a small molecular compound that can be decomposed into nitrides and sulfides at high temperatures. In this work, sulfur- and nitrogen-codoped nonprecious carbon-based bifunctional oxygen catalyst SnFe/SNC_T (where T refers to pyrolysis temperatures such as 750, 850, and 950 °C) was synthesized using thiourea and g-C_3_N_4_ as sulfur and nitrogen sources, respectively, and pyrolyzed with tin dichloride and ferric chloride hexahydrate at high temperature. The bifunctional ORR/OER performance and durability measured in 0.1 M KOH solution were excellent. A remarkable ORR performance is also demonstrated in the single-cell test of the anion exchange membrane fuel cell. Despite the widespread use of S and N codoping in carbon-based electrocatalysts, the present work distinguishes itself by integrating S and N heteroatoms with a dual-metal Sn–Fe system, anchored in a bamboo-like tubular carbon network derived from g-C_3_N_4_ and thiourea. This combination introduces synergistic effects between Fe–N_*x*_/Fe–S_*x*_ moieties and intermetallic Fe_3_SnC/FeS domains, enabling improved intrinsic activity and enhanced durability. Unlike previous codoped or Fe-only catalysts, the inclusion of Sn alters the electronic environment of Fe active sites, as supported by comparative control experiments, XPS, and XAS analysis.

## 2 Experimental Section

### 2.1 Chemicals and Reagents

Melamine (C_3_H_6_N_6_, ≥99%), Tin­(II) chloride dihydrate (SnCl_2_·2H_2_O, ≥98%), Iron­(III) chloride hexahydrate (FeCl_3_·6H_2_O, ≥99%), and Thiourea (CH_4_N_2_S, ≥99%) were procured from Acros Organics. All chemicals were of analytical grade (unless otherwise specified) and used as received without further purification.

### 2.2 Preparation of SnFe/SNC_T

SnFe/SNC_*T* catalysts were synthesized using a previously reported procedure with a slight modification.^5^ The catalyst synthesis is schematically illustrated in Figure 1. Briefly, Melamine was pyrolyzed in a muffle furnace at 550 °C for 4 h at a heating rate of 5 °C/min, yielding yellow g-C3N4. Subsequently, 0.265 g of SnCl_2_·2H_2_O (1.4 mmol) and 0.76 g of FeCl_3_·6H_2_O (2.8 mmol) were dissolved in 50 mL of ethanol, and then 2 g of g-C_3_N_4_ and 1 g of Thiourea (CH_4_N_2_S) were added to the solution. The mixture was stirred at 50 °C for 16 h to ensure that the metal ions formed complexes with thiourea and were thoroughly adsorbed onto g-C_3_N_4_. The solvent was then evaporated to obtain the dried powder. Then, the dried powder precursor was pyrolyzed at 750, 850, and 950 °C for 2 h under a nitrogen atmosphere, with a heating rate of 5 °C/min, to obtain the sulfur- and nitrogen-codoped catalyst SnFe/SNC_*T* (where *T* is the pyrolysis temperature of 750, 850, or 950 °C).

**Figure 1:**
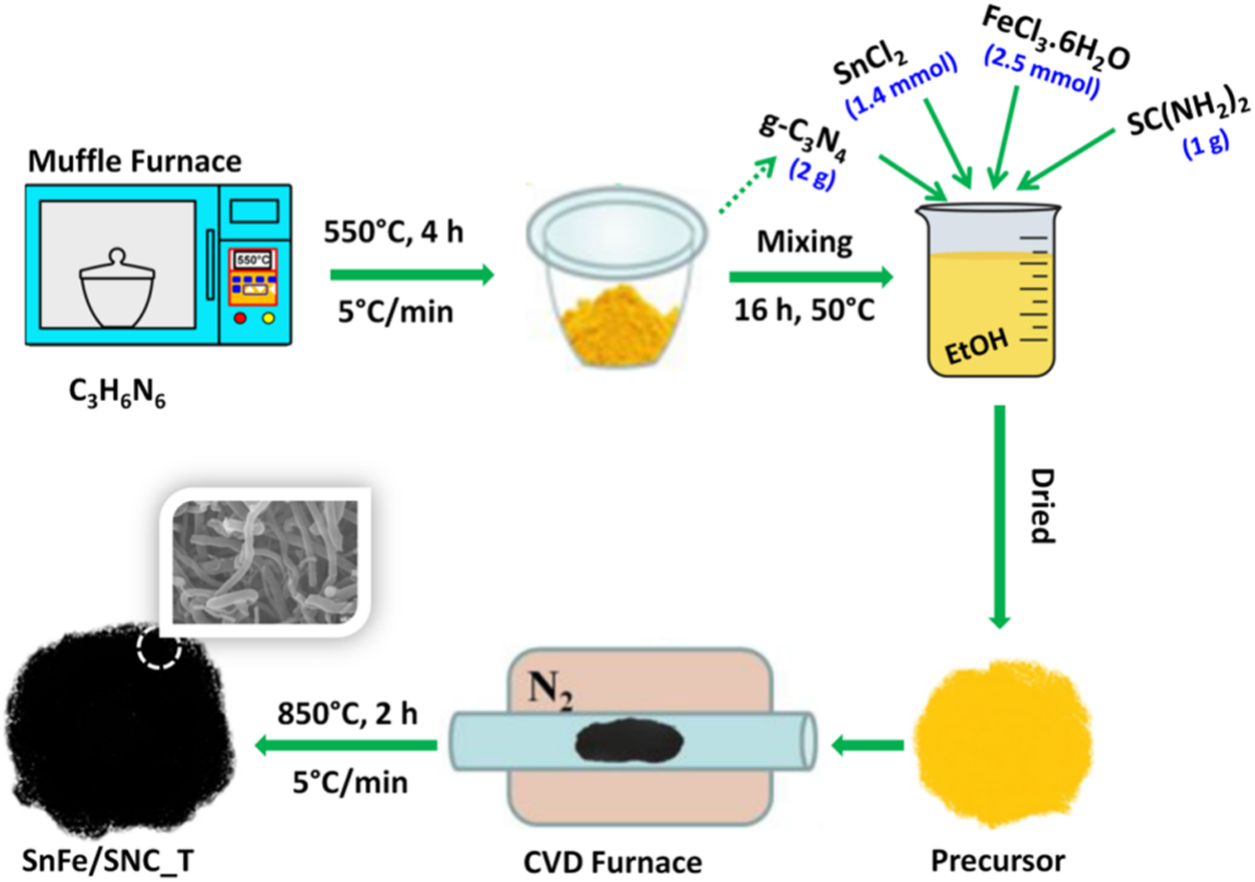
Schematics showing the preparation of SnFe/SNC_*T* (*T* = 750, 850, and 950 °C) catalyst.

### 2.3 Characterizations

Crystallographic information was obtained using X-ray diffraction (XRD; Bruker D2-Phaser, Cu Kα radiation). Raman spectroscopy (Horiba Jobin Yvon iHR550) was used to assess graphitization and defects. Morphological and nanostructural analyses were performed using field-emission scanning electron microscopy (FESEM; JEOL JSM-6500F) and transmission electron microscopy (TEM; JEOL JEM-2100) equipped with energy-dispersive X-ray spectroscopy (EDS). Brunauer–Emmett–Teller (BET) specific surface area and pore size distribution were determined from N_2_ physisorption isotherms at 77 K (Micromeritics ASAP 2420) after sample degassing. Electron paramagnetic resonance (EPR) spectroscopy (Bruker ESR5000) was used to identify potential vacancies. X-ray photoelectron spectroscopy (XPS; ULVAC-PHI Inc. PHI 5000 VersaProbe III, Al Kα X-ray source) was employed to determine the chemical composition and oxidation states of elements (C 1s, N 1s, O 1s, S 2p, Sn 3d, Fe 2p), with binding energies calibrated to the C 1s peak at 284.8 eV. Fe K-edge X-ray absorption spectroscopy (XAS) was performed at beamline 17C1 of the NSRRC (Hsinchu, Taiwan) using a multipole wiggler source (2.7 keV critical energy) with a 1.5 GeV storage ring and 120–200 mA beam current. Spectra were recorded in transmission mode at room temperature with gas-filled ionization chambers. Catalyst powders were pressed into stainless-steel holders, and Fe foil was used for energy calibration. Data processing, including background subtraction, normalization, and Fourier transformation, was carried out using Athena.

### 2.4 Electrochemical Analysis

All electrochemical measurements were performed at room temperature (approximately 25 °C) using a Biologic VSP potentiostat/galvanostat in a standard three-electrode cell configuration.

#### 2.4.1 Oxygen Reduction Reaction (ORR) Measurements

A 0.1 M potassium hydroxide (KOH) aqueous solution served as the electrolyte for ORR studies. A graphite rod was used as the counter electrode, and a saturated calomel electrode (SCE, Hg/Hg_2_Cl_2_ in saturated KCl) acted as the reference electrode. All potentials were converted to the reversible hydrogen electrode (RHE) scale using the Nernst equation: *E*(RHE) = *E*(SCE) + 0.241 V + 0.059 × pH. The pH of 0.1 M KOH was taken as 13. The working electrode was prepared as follows: 5 mg of the catalyst was dispersed in a mixture of 1.4 mL of deionized water and 0.608 mL of ethanol. The mixture was ultrasonicated to form a homogeneous ink. A specific volume of this ink was then drop-cast onto a polished rotating ring-disk electrode (RRDE, Pine Research Instrumentation, model AFE6R2GCAU, glassy carbon disk area = 0.2376 cm^2^, Pt ring) to achieve a catalyst loading of 200 μg cm^–2^. After deposition, 5 μL of 0.05 wt % Nafion solution (diluted from a 5 wt % stock solution) was dropped onto the catalyst layer as a binder, and the electrode was allowed to air-dry.

Before ORR measurements, the 0.1 M KOH electrolyte was saturated with high-purity O_2_ by bubbling for at least 30 min. Cyclic voltammetry (CV) was performed in the O_2_-saturated electrolyte at a scan rate of 10 mV s^–1^ within a potential window of 0 to 1.2 V vs RHE. Linear sweep voltammetry (LSV) for ORR was subsequently recorded at a scan rate of 10 mV s^–1^ with an electrode rotation speed of 1600 rpm under a continuous O_2_ flow. The electron transfer number (*n*) and hydrogen peroxide yield (% HO_2_^–^) were calculated from the RRDE data using the following equations

1 $$n=4\left(\frac{I_{d}}{I_{d}+\frac{I_{r}}{N}}\right)$$

2 $$\%{\mathrm{HO}}_{2}^{-}=\left(\frac{2I_{r}/N}{I_{d}+\frac{I_{r}}{N}}\right)\times 100\%$$

where *I*_d_ is the disk current, *I*_r_ is the ring current, and *N* is the current collection efficiency of the Pt ring (*N* = 0.361, as provided by the manufacturer). Electrochemical impedance spectroscopy (EIS) was performed at 0.85 V vs RHE under steady-state conditions over a frequency range of 200 kHz to 10 mHz with an amplitude of 10 mV.

#### 2.4.2 Oxygen Evolution Reaction (OER) Measurements

OER activity was evaluated in an N_2_-saturated 1 M KOH solution. A gold rotating disk electrode (Au RDE, Pine Research Instrumentation, model AFE3T050 AU, disk area = 0.196 cm^2^) served as the working electrode substrate. The working electrode for OER was prepared by dispersing 5 mg of the catalyst in a mixture of 980 μL deionized water, 980 μL absolute ethanol, and 40 μL 5 wt % Nafion solution (resulting in an approximate Nafion concentration of 0.1 wt % in the ink relative to the solvents). The mixture was ultrasonicated. Catalyst inks were then drop-casted onto the Au RDE to achieve loadings of 200, 300, and 400 μg cm^–2^ and allowed to dry under ambient conditions. For comparison, a benchmark OER catalyst was prepared using a mixture of commercial RuO_2_ (Aladdin) and Vulcan XC-72R carbon black (2:8 mass ratio) and had the same catalyst loadings on the Au RDE, following a similar ink preparation and deposition procedure. The 85% *iR* compensation was applied to the electrochemical OER measurement as

3 $$E_{\mathrm{RHE},\mathrm{iR}}=E_{\mathrm{RHE}}-\mathrm{iR}\times 0.85$$

### 2.5 AEMFC Single-Cell Test

All single-cell measurements were conducted in an actual anion exchange membrane fuel cell (AEMFC) configuration. A Sustainion X37–50 RT membrane, pretreated in 1 M KOH, was used and assembled into a membrane electrode assembly (MEA) with a fixed geometric area of 2.7 cm × 2.7 cm. The MEA comprised a spray-coated SnFe/SNC_850 cathode catalyst and a commercial Pt/C anode catalyst. The membrane pretreatment was performed according to previously reported procedures.^15^ Briefly, the membrane was immersed in 1 M KOH solution for 48 h, with the solution changed every 24 h to convert the Br^–^ or Cl^–^ ions on the membrane into OH^–^ ions. The membrane was then immediately rinsed with deionized water and was ready for use. To prepare the electrodes, the as-synthesized cathode catalyst was mixed with ionomer at a 3:1 mass ratio and dispersed in methanol under sonication for 4 h. The catalyst ink was applied to the carbon fabric with a microporous layer (MPL) using a spray coater to form the cathode, with a catalyst loading of 0.6 mg cm^–2^. A similar procedure was followed to prepare the anode using the 20% Pt/C commercial. The spray-coated electrodes were dried in a vacuum oven at 50 °C side by side. The anode and cathode were also pretreated by being submerged in 1 M KOH solution for 48 h, with the solution changed every 24 h, rinsed with DI water, and then ready for assembly. Finally, the AEMFC performance was measured at the fuel cell test station (Tension Energy, Inc.) at 65 °C under no back pressure to obtain the polarization curve. The currents and cell voltages were recorded once the steady state was reached. Both hydrogen and oxygen flow rates were 200 standard cubic centimeters per minute (SCCM) with 100% humidity at 50 °C before entering the MEA device.

### 2.6 DFT Calculations

Density functional theory (DFT) calculations were performed using the Vienna Ab initio Simulation Package (VASP) with the projector augmented-wave (PAW) method.^30^ The generalized gradient approximation (GGA) was adopted using the Perdew–Burke–Ernzerhof (PBE) exchange–correlation functional.^31^ The plane-wave basis set was truncated at a kinetic energy cutoff of 520 eV. Dispersion interactions were taken into account using Grimme’s DFT-D3 method with Becke–Johnson damping.^32^ To represent the nitrogen-rich carbon framework derived from g-C_3_N_4_ during pyrolysis, a 2 × 2 heptazine-based g-C_3_N_4_ supercell containing 56 atoms was employed as the starting structural motif. Dopant configurations including Fe-, Sn-, S-, Fe–S-, Sn–S-, and Fe–Sn–S-doped structures were generated by substituting selected atoms within the same supercell. In the experimental catalyst, g-C_3_N_4_ serves as a sacrificial precursor that transforms into an N-doped carbon framework containing embedded Sn–Fe intermetallic domains after carbonization. Since modeling the full nanotube-derived carbon structure with intermetallic nanoparticles would require prohibitively large computational cells, the g-C_3_N_4_-derived motif was adopted to represent the local nitrogen coordination environment surrounding the active metal sites. Such simplified models are widely used in theoretical studies of heteroatom-doped carbon electrocatalysts to investigate the electronic structure and adsorption behavior of oxygen intermediates at representative catalytic centers, while maintaining computational feasibility.^33^ The model, therefore, captures the local electronic modulation induced by Fe, Sn, and S dopants rather than reproducing the full structural complexity of the experimental catalyst. The structures were visualized using iRASPA software.^34^ A vacuum spacing of 18 Å was applied along the perpendicular direction to eliminate interactions between periodic images. Spin-polarized calculations were carried out throughout. An initial magnetic moment of 4 μ_B_ was assigned to Fe atoms, while zero initial moments were set for all other atoms. Structural relaxations were performed until the maximum force on each atom fell below 0.02 eV/Å and the energy difference between successive electronic steps was less than 10^–6^ eV. The Brillouin zone was sampled using a Γ-centered 3 × 3 × 1 Monkhorst–Pack *k*-point mesh. For density-of-states plots, a denser k-mesh was employed. The free energy diagrams were constructed using the computational hydrogen electrode model. All calculations were performed at *U* = 0 V versus the reversible hydrogen electrode, where the free energies of proton–electron pairs (H^+^ + e^–^) are referenced to 1/2 H_2_ (g). The reaction free energies for the OER intermediates (*OH, *O, *OOH) were calculated as Δ*G* = Δ*E*_DFT_ + Δ*E*_ZPE_ – *T*Δ*S*, with zero-point energies (ZPE) and entropies (*S*) obtained from vibrational frequency calculations at 298.15 K.

## 3 Results and Discussion

As depicted in the synthesis scheme (Figure 1), the precursor from g-C_3_N_4_, thiourea, tin dichloride, and ferric chloride was pyrolyzed at 750, 850, and 950 °C to obtain the catalyst SnFe/SNC_*T* (where *T* is the pyrolysis temperature 750, 850, and 950 °C). Before synthesizing the SnFe/SNC_*T* catalyst, we optimized the heteroatom-doped carbon framework. Briefly, we first investigated the influence of the g-C_3_N_4_ (N precursor) loading on the ORR activity of bare N-doped carbon (NC). NC samples were synthesized using 1, 1.5, 2, and 2.5 g of g-C_3_N_4_ at 850 °C, and their ORR polarization curves are shown in Figure S1a of the Supporting Information. The NC prepared with 2 g of g-C_3_N_4_ exhibited the most positive onset potential and the highest limiting current density, indicating an optimal balance among nitrogen content, electrical conductivity, and porosity.^35^ At lower loadings (1–1.5 g), ORR activity was limited, likely due to insufficient N incorporation into the carbon matrix, resulting in a low density of active pyridinic and graphitic N sites.^35^ Conversely, excessive g-C_3_N_4_ (2.5 g) resulted in a noticeable drop in ORR performance. This phenomenon is commonly associated with either a nonuniform distribution of the precursor, leading to aggregation and pore blockage that hinder mass transfer kinetics, or an undesirable change in the final carbon structure, such as excessive graphitization or the formation of less active N-species.^36,37^ With the g-C_3_N_4_ amount fixed at 2 g, we next optimized the sulfur precursor by varying the thiourea content (0.25, 0.5, 0.75, 1.0, and 1.25 g) to prepare S,N-*co*-doped carbons (SNC). As shown in Figure S1b of the Supporting Information, the SNC sample obtained with 1.0 g thiourea exhibits the best ORR performance, featuring both the most positive onset potential and the highest kinetic current density. Lower thiourea loadings result in insufficient S incorporation, while higher amounts (≥1.25 g) lead to diminished activity, which may be related to excessive sulfur species and structural defects that compromise conductivity.^38^ These optimization results demonstrate that 2 g g-C_3_N_4_ and 1 g thiourea provide an optimal S,N codoping level and were therefore selected as the standard conditions for the subsequent synthesis of SnFe/SNC_*T* catalysts. Similarly, the Sn:Fe molar ratio was systematically optimized based on ORR performance investigations. Four Sn/Fe molar ratios (1:0.5, 1:1, 1:2, and 1:3) of the SnFe/SNC synthesized at 850 °C were investigated for preliminary ORR performance based on the linear sweep voltammetry (LSV) in 0.1 M KOH. As it is observed in Figure S1c of Supporting Information, the best ORR performance was obtained at the 1:2 (Sn/Fe) molar ratio, and this was used for further investigations. A further increase in the Sn:Fe molar ratio declines the ORR catalytic activity, potentially due to increased nanoparticle aggregation, which limits atomic dispersion within the carbon matrix and suppresses the formation and accessibility of catalytically active sites.

SEM imaging of SnFe/SNC_850 (Figure 2a) revealed a distinct bamboo-like tubular morphology, with tube diameters consistently in the range of 88.5 to 95.7 nm (Figure S2a of Supporting Information). These carbon nanotubes hosted numerous nanoparticles, which were observed both encapsulated within the tubes and adsorbed on their external surfaces (Figure 2b, yellow circles). SEM-EDS analysis confirmed the significant presence of these nanoparticles (Figure S2b of Supporting Information). TEM analysis (Figure 2c) further corroborated the typical bamboo-like tubular structure, clearly showing nanoparticles confined within the bamboo cavities, particularly at the nodes. This unique architecture, attributed to a vapor–liquid–solid (VLS) growth mechanism on molten metal^39^ and facilitated by Fe doping,^40^ enhances mass transfer and, consequently, electrocatalytic performance.^41^ In the Fe-driven VLS growth mechanism, the Fe nanoparticles formed during pyrolysis catalyze the anisotropic carbon deposition. Upon decomposition of the g-C_3_N_4_, a structured nitrogen-rich carbon source, gaseous carbonaceous species are absorbed by molten Fe particles. Once supersaturation is reached, carbon precipitates anisotropically, forming segmented tubular carbon. The bamboo-like segmentation arises from periodic fluctuations in carbon solubility or surface tension at the Fe droplet, as previously reported.^40,42^ The presence of Sn further modifies the catalytic behavior and melting point of Fe, supporting this morphology by stabilizing intermediate intermetallic phases (e.g., Fe_3_SnC), which tune nucleation and growth dynamics. S and N codoping originates from the decomposition of thiourea (S source) and g-C_3_N_4_ (N and C source) during pyrolysis. Thus, the synergistic combination of g-C_3_N_4_ as a structured carbon/nitrogen source and Fe-driven VLS growth enables the controlled formation of bamboo-like tubular carbon architectures.

**Figure 2:**
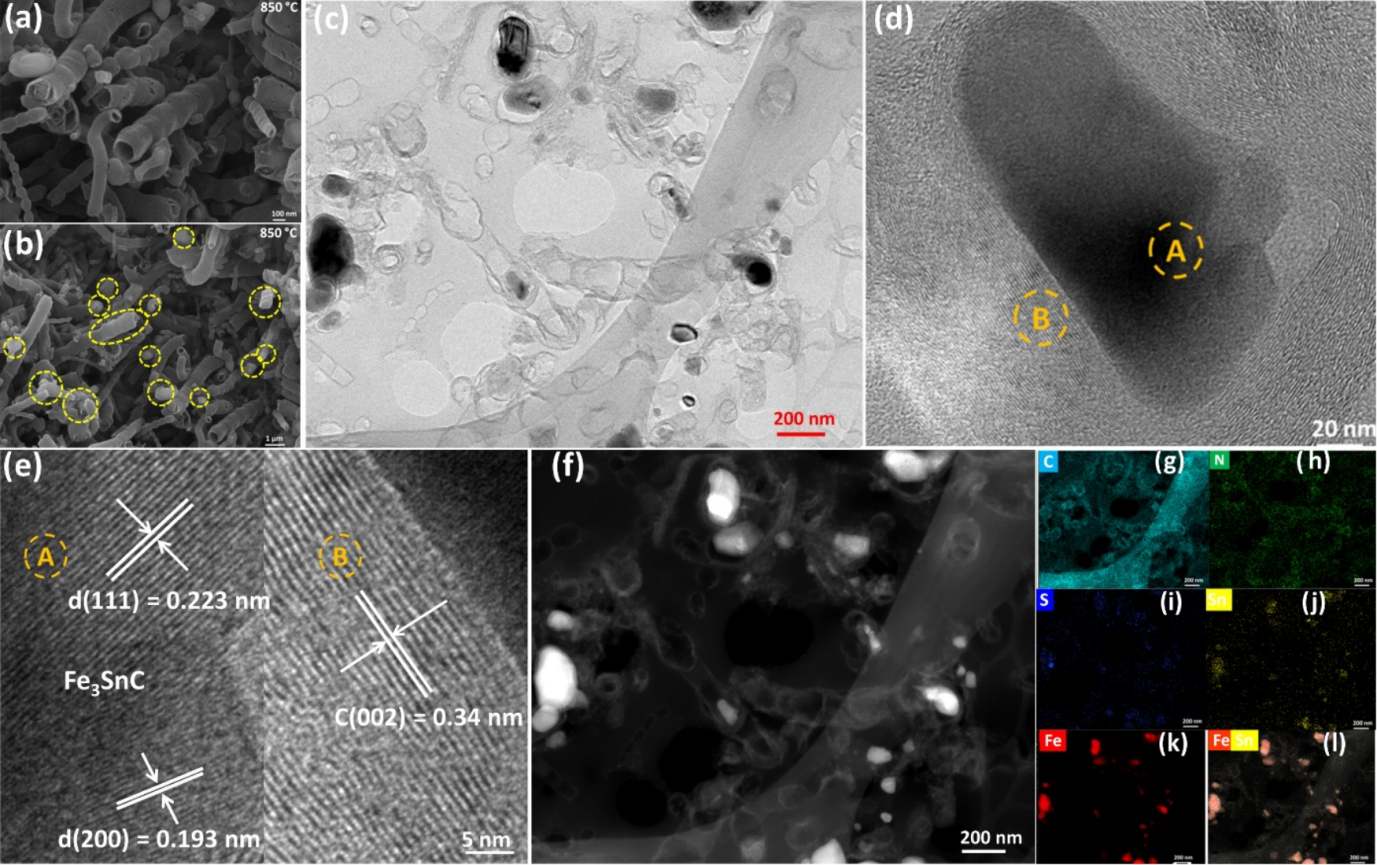
(a) SEM image of SnFe/SNC_850, (b) SEM image of SnFe/SNC_850 showing the nanoparticles marked in yellow circles, (c) TEM, (d, e) HRTEM, (f) HAADF-STEM images of SnFe/SNC_850 and the corresponding EDS elemental mappings of (g) C, (h) N, (i) S, (j) Sn, (k) Fe, (l) Fe–Sn.

High-resolution TEM (HRTEM) provided detailed structural insights. As shown in Figure 2d, graphitic carbon layers (e.g., position B) encapsulate nanoparticles (e.g., position A). These carbon layers exhibited an interplanar distance of 0.34 nm, characteristic of the (002) plane of graphitic carbon (Figure 2e, position B). Crucially, the encapsulated nanoparticles displayed well-resolved lattice fringes with interplanar distances of 0.223 and 0.193 nm (Figure 2e, position A), corresponding to the (111) and (200) planes of cubic Fe_3_SnC, respectively.

HAADF-STEM imaging (Figure 2f) further elucidated the SnFe/SNC_850 structure, where bright spots, representing nanoclusters, were predominantly located within the cavities at the nodes and ends of the tubes. Corresponding elemental mapping (Figure 2g–k) revealed a uniform N, S, and Sn distribution throughout the carbon matrix. In stark contrast, Fe was significantly enriched within these nanocluster regions. Fe–Sn comapping (Figure 2l) confirmed that these confined nanoparticles are coexisting Fe and Sn, consistent with the Fe_3_SnC phase. The elemental maps also unequivocally verified the successful doping of S and N atoms into the carbon matrix. Such heteroatom doping is widely recognized for redistributing charge density within the carbon framework, thereby creating catalytically active sites crucial for ORR in carbon-based catalysts.^4,5^

The pyrolysis temperature is critical in tailoring the bamboo tubular morphology and, consequently, the catalyst’s performance. While tubes began to form at 750 °C (Figure S3a of the Supporting Information), well-defined bamboo-like tubular networks were achieved at 850 °C (Figure S3b of the Supporting Information). However, increasing the temperature to 950 °C (Figure S3c of Supporting Information) leads to the disappearance of bamboo nodes and the formation of broken tubes with larger diameters and smoother surfaces, indicating structural instability of the desired bamboo morphology at excessively high temperatures.

X-ray diffraction (XRD) analysis was employed to investigate the crystallographic structures of various catalysts pyrolyzed at different temperatures, as shown in Figure 3a. All samples displayed a broad diffraction peak around 26°, characteristic of the (002) plane of graphitic carbon. The patterns revealed the presence of metallic Sn (e.g., characteristic reflections at 30.7°, 32.1°; PDF#01–0926) and metallic Fe (PDF#01–1252). More significantly, distinct peaks corresponding to the intermetallic Fe_3_SnC phase (e.g., at 23.0°, 32.7°, 40.4°; PDF#89–7284) and FeS (e.g., at 29.8°, 33.7°; PDF#89–1950) were identified, indicating complex phase formation during pyrolysis. The formation of the Fe_3_SnC intermetallic phase is likely due to solid-state reactions between zerovalent Fe and Sn with carbon derived from precursor decomposition, involving diffusion, alloying, and subsequent carbon intercalation. The presence of Fe_3_SnC signifies successful Fe–Sn alloying at high temperatures. It is crucial as it can modulate the electronic structure, improve the anchoring of active Fe–N_*x*_ and Fe–S_*x*_ sites, and provide a robust framework for charge transport and catalyst stability. Thus, XRD confirms that the SnFe/SNC_*T* catalysts consist of metallic Sn, Fe, Fe_3_SnC, and FeS phases embedded within a graphitic carbon matrix.

**Figure 3:**
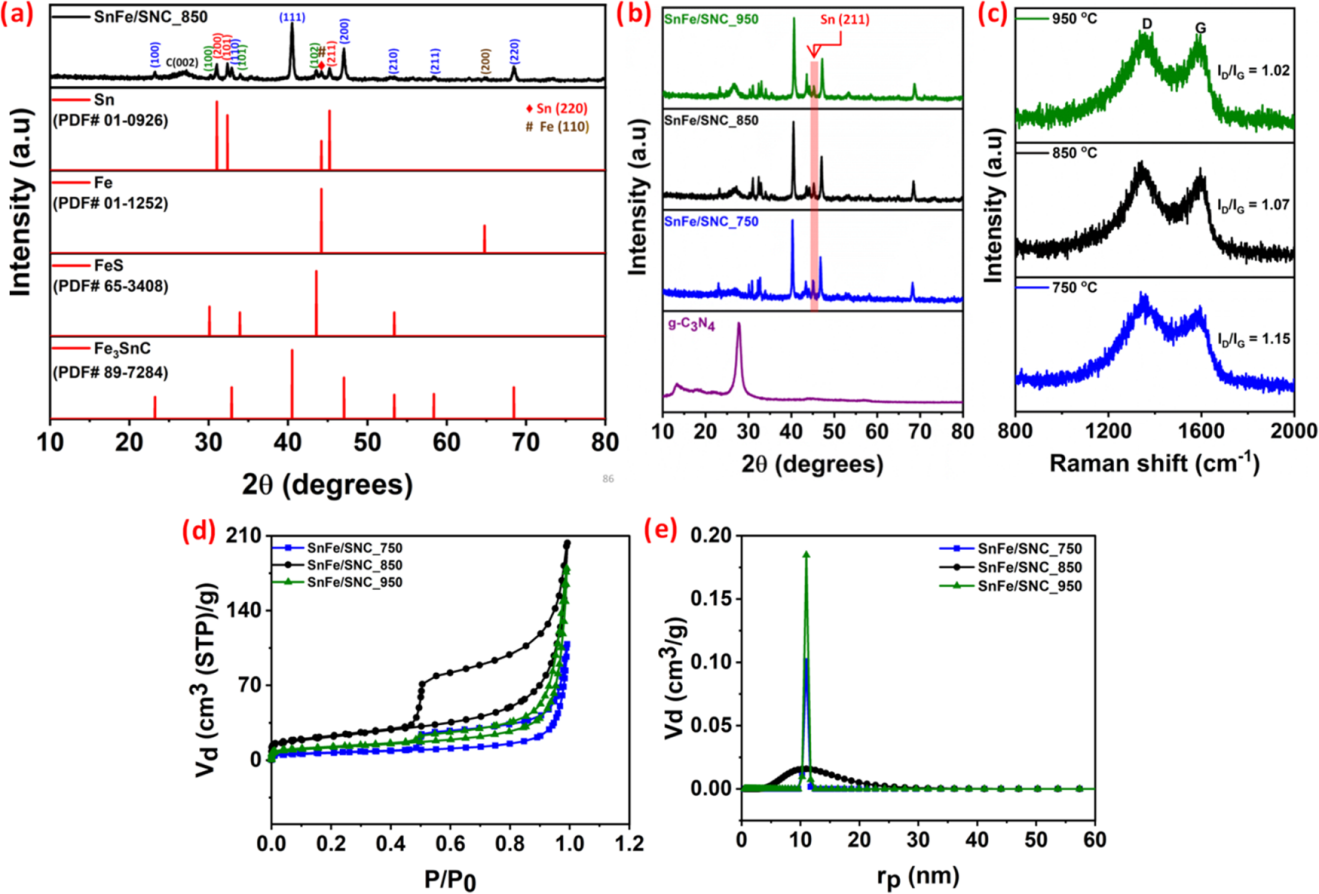
(a, b) XRD spectra, (c) Raman spectra, (d) N-adsorption/desorption isotherm, and (e) pore size distribution of SnFe/SNC_*T* (*T*: 750, 850, and 950 °C).

The influence of pyrolysis temperature was evident in the carbon structure and the metallic phases (Figure 3b). At 750 °C, the initial transformation of g-C_3_N_4_ into nitrogen-doped carbon (NC) with low graphitization was observed, alongside the crystallization of adsorbed metal species into metallic Sn, Fe, and nanoclusters of Fe_3_SnC and FeS. Increasing the temperature to 950 °C enhanced the graphitization degree of the NC and the crystallinity of these composite phases. Concurrently, the intensity of the metallic Sn peaks decreased at 950 °C, likely due to Sn evaporation at high temperatures,^43^ a trend consistent with EDS elemental analysis (Table S1 of the Supporting Information). This increased graphitization at higher temperatures was further corroborated by Raman spectroscopy (Figure 3c), where the *I*_D_/*I*_G_ ratio decreased from SnFe/SNC_750 to SnFe/SNC_950, signifying a higher degree of graphitic order and fewer defects. These metallic phases (single and composite) are known to facilitate the graphitization of nitrogen-containing precursors and heteroatom doping.^44^ Notably, XRD detection of metal sulfides (FeS) suggests potential active sites for the oxygen evolution reaction (OER).^45^

Nitrogen adsorption–desorption analysis was performed to evaluate the catalysts’ porosity. All SnFe/SNC_*T* samples exhibit type IV isotherms with H3 hysteresis loops (Figure 3d), characteristic of mesoporous materials. Their pore size distributions, derived from these isotherms (Figure 3e), were predominantly centered in the mesopore range. Interestingly, SnFe/SNC_850 shows a somewhat broader mesopore distribution. Crucially, SnFe/SNC_850 possessed the largest BET-specific surface area (76.9 m^2^ g^–1^) compared to SnFe/SNC_950 (41.5 m^2^ g^–1^) and SnFe/SNC_750 (23.6 m^2^ g^–1^). This superior surface area, combined with its mesoporous structure, is expected to expose more catalytically active sites and facilitate O_2_ transport, enhancing ORR and OER performance.^46^

To further elucidate the elemental composition, valence states, and bonding configurations of the synthesized catalysts, X-ray photoelectron spectroscopy (XPS) was conducted. The survey spectrum of SnFe/SNC_850 (Figure S4 of the Supporting Information) confirmed the presence of C, N, S, Fe, and Sn. A minor oxygen peak was also observed, likely due to partial surface oxidation during synthesis.^16^ High-resolution XPS spectra for the C 1s, N 1s, S 2p, Fe 2p, and Sn 3d regions of SnFe/SNC_850 are presented in Figure 4a–4e, providing detailed insights into their chemical states.

**Figure 4:**
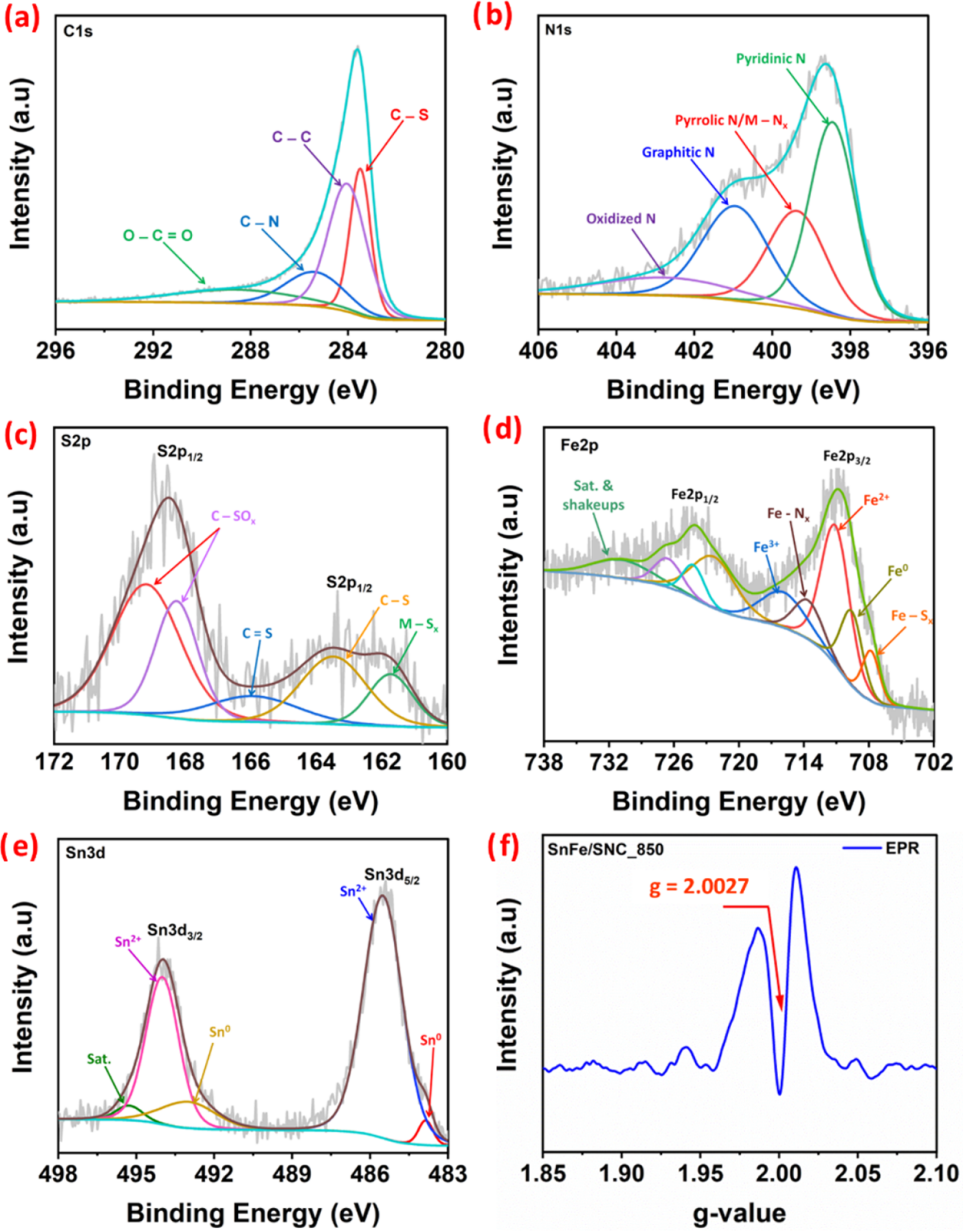
High-resolution XPS spectra (a) C 1s, (b) N 1s, (c) S 2p, (d) Fe 2p, and (e) Sn 3d of SnFe/SNC_850; (f) EPR spectrum of SnFe/SNC_850.

The high-resolution C 1s spectrum (Figure 4a) was deconvoluted into peaks corresponding to C–S (283.5 eV), C–C (sp^2^ carbon, 284.05 eV), C–N (285.45 eV), and O–C=O (288.8 eV) configurations.^6,47^ The distinct C–N and C–S peaks confirm the successful incorporation of nitrogen and sulfur into the carbon matrix. The N 1s spectrum (Figure 4b) revealed several nitrogen species: pyridinic N (398.4 eV), pyrrolic N/M-N*_x_* (399.4 eV, M predominantly Fe), graphitic N (401.0 eV), and oxidized N (402.9 eV) with the pyridinic N showing the highest relative concentration (Table S2 of the Supporting Information).^4^ Among these, pyridinic and graphitic N are significant because they can modify the charge distribution of the carbon material, facilitate the 4-electron oxygen reduction pathway, and provide anchoring sites for metal atoms.^4,13^ Similarly, the S 2p spectrum (Figure 4c) showed signals for M-S*_x_* (161.7 eV, M predominantly Fe), C–S (163.5 eV), C=S (165.8 eV), and oxidized sulfur (C–SO_*x*_ at 168.3 and 169.2 eV).^4,28^ The relative concentrations of the C–S and oxidized S species (e.g., C–SO_*x*_) are shown in Table S2 of Supporting Information, with the C–SO_*x*_ species showing dominance. The codoping of these N and S heteroatoms is believed to redistribute charge density synergistically, creating highly active ORR/OER sites within the carbon framework.^4^ These S and N species play distinct catalytic roles, with pyridinic-N and C–S structures enhancing *O_2_ adsorption and electron transfer for ORR. At the same time, graphitic-N and oxidized sulfur improve *OH/*O intermediate stabilization for OER.^48,49^ The synergy between S,N codoping and the tubular morphology arises from the combination of electronic modulation (via heteroatom doping) and accelerated mass/electron transport (via the 1D hollow architecture).^40^ The interconnected tubular network offers high surface area, shortened ion diffusion paths, and accessible active sites, leading to enhanced bifunctional electrocatalytic performance.^40^

The high-resolution Fe 2p spectrum (Figure 4d) provided further evidence for metal-heteroatom interactions and iron’s valence states. Peaks at 707.9 and 713.85 eV were assigned to Fe–S_*x*_ and Fe–N_*x*_ configurations, respectively, corroborating the N 1s and S 2p findings. Additionally, signals for Fe^0^ (709.75 eV), Fe^2+^ (711.25 eV), and Fe^3+^ (716.25 eV) were identified, with Fe^2+^ being the dominant oxidation state. The density of Fe–N*_x_* and Fe–S*_x_* sites was determined based on the relative peak areas of the deconvoluted M-N_*x*_ and M-S_*x*_ coordinations in the high-resolution N 1s and S 2p XPS spectra, respectively (Table S2 of the Supporting Information), and compared with the density of Fe–N_*x*_ and Fe–S_*x*_ coordinations determined based on their relative peak areas in the high-resolution Fe 2p (Table S3 of the Supporting Information), enabling semiquantitative evaluation of these species. In both cases, the relative % concentrations indicate that Fe–N*_x_* coordination is predominant in SnFe/SNC_850, accompanied by a significant contribution from Fe–S*_x_* moieties (Tables S2 and S3 of Supporting Information). The Sn 3d spectrum (Figure 4e) displayed doublets corresponding to Sn^0^ (483.85 eV for Sn 3d_5/2_ and 493.05 eV for Sn 3d_3/2_) and Sn^2+^ (485.55 eV for Sn 3d_5/2_ and 494.0 eV for Sn 3d_3/2_) species.^13,50^ The XPS results indicate the presence of Fe^0^ and Sn^0^, consistent with the formation of Fe_3_SnC bimetallic nanoparticles. Furthermore, detecting Fe^2+^/Fe^3+^ states, along with the N 1s and S 2p data, confirms the existence of M-N_*x*_ and M-S_*x*_ moieties. These M-N_*x*_ and M-S_*x*_ sites are considered crucial active centers for ORR, while M-S_*x*_ can also contribute to OER activity, underpinning the bifunctional electrocatalytic performance of SnFe/SNC_850.^5,13^ Electron Paramagnetic Resonance (EPR) spectroscopy provided further insights into defect structures (Figure 4f). SnFe/SNC_850 exhibited a distinct signal at a *g*-value of approximately 2.0027, characteristic of N or S vacancies within the carbon matrix.^51^ The presence of these vacancies can further modulate the electronic properties and create additional active sites, thereby enhancing the catalytic activity of SnFe/SNC_850.

Fe K-edge X-ray absorption near-edge structure (XANES) and extended X-ray absorption fine structure (EXAFS) analysis were performed to gain deeper insight into the electronic state and local coordination of Fe in SnFe/SNC_850. As shown in the XANES spectra (Figure 5a), the absorption edge of SnFe/SNC_850 lies between those of Fe foil and FeO, suggesting an average Fe valence state between 0 and +2, in excellent agreement with XPS results. This intermediate oxidation state indicates a balance between electron-rich Fe centers (favorable for O_2_ activation) and partially oxidized species (which can stabilize oxygenated intermediates).^52,53^ Doped heteroatoms are known to modulate the electronic state of Fe-based sites and improve ORR activity.^54^ The Fourier-transformed EXAFS spectrum (Figure 5b) displays distinct peaks at ∼1.49 Å and ∼1.79 Å, corresponding to Fe–N_*x*_ and Fe–S_*x*_ coordination, respectively. The coexistence of these coordination environments confirms the successful embedding of atomically dispersed Fe sites within N/S-doped carbon, where N provides strong anchoring and electron delocalization, while S introduces electronic asymmetry that can optimize adsorption energies of ORR/OER intermediates; synergistic effects of S/N codoping on these aspects have been reported in similar systems.^55,56^ In addition, a peak at ∼2.4 Å, slightly shifted compared to the Fe foil, suggests the presence of Fe–Fe metallic coordination, which may originate from confined Fe_3_SnC/FeS nanophases encapsulated within the carbon. These dual features imply that the SnFe/SNC_850 catalyst benefits simultaneously from isolated Fe–N_*x*_/S_*x*_ moieties (highly active and selective sites for ORR) and metallic phases that enhance conductivity and potentially improve OER kinetics. Together, these structural insights corroborate the superior bifunctional activity and durability of SnFe/SNC_850 by linking its atomic-scale configuration to its electrochemical performance.

**Figure 5:**
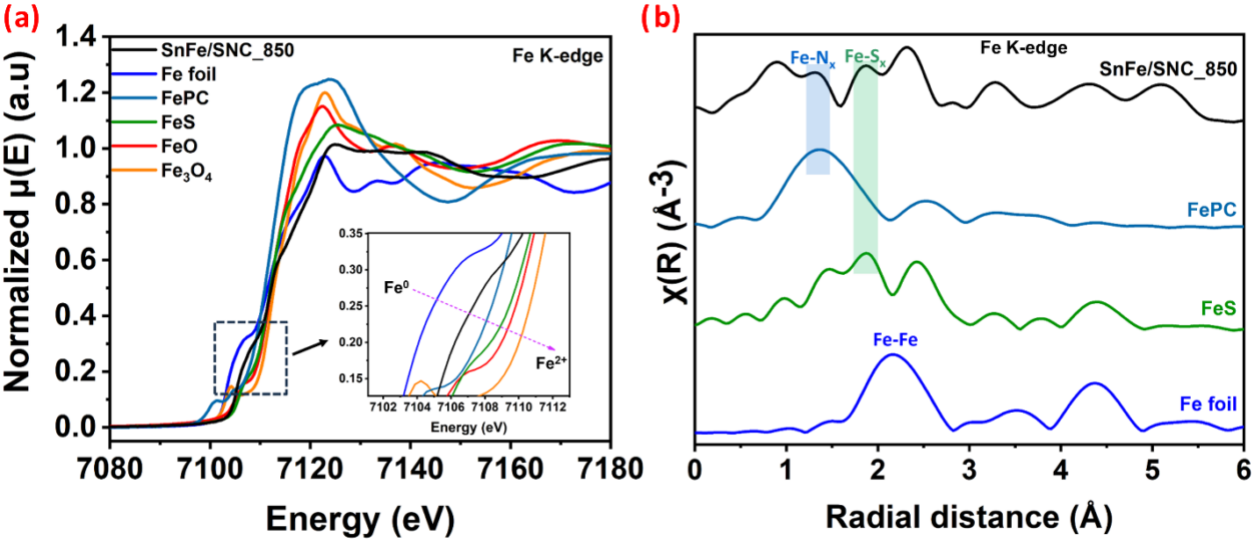
XAS normalized Fe K-edge (a) XANES and (b) EXAFS spectra of SnFe/SNC_850 and standards.

The synthesized catalysts’ oxygen reduction reaction (ORR) activities were evaluated in a 0.1 M KOH electrolyte using a three-electrode system with a Rotating Ring-Disk Electrode (RRDE) and benchmarked against commercial 20% Pt/C. Cyclic Voltammetry (CV) curves in O_2_-saturated 0.1 M KOH (Figure 6a) revealed distinct O_2_ reduction peaks at 0.81, 0.87, and 0.82 V vs RHE for SnFe/SNC_750, SnFe/SNC_850, and SnFe/SNC_950, respectively, indicating that all synthesized catalysts possess ORR activity. SnFe/SNC_850 exhibits a more positive O_2_ reduction peak potential (0.87 V) than 20% Pt/C, signifying its superior intrinsic ORR activity. This superior activity implies a lower overpotential requirement for ORR, potentially attributed to more efficient electron transfer, optimized binding energies for reaction intermediates, or synergistic effects from its unique composition. Such characteristics are highly desirable for practical applications like fuel cells, where high activity at minimal overpotential is crucial.

**Figure 6:**
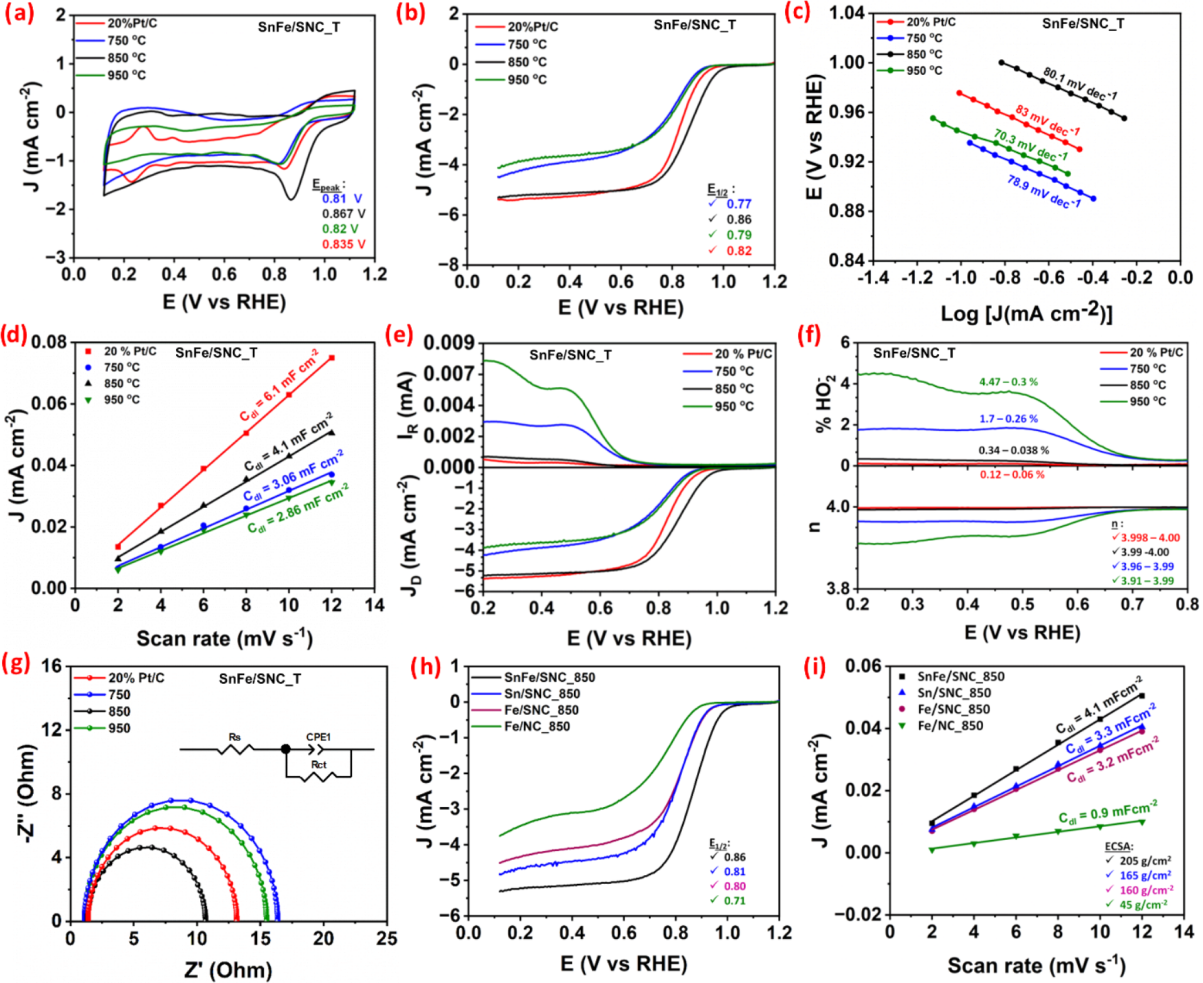
(a) Cyclic voltammetry (CV), (b) linear sweep voltammetry (LSV), (c) Tafel plot, (d) ECSA, (e) *J*_D_ vs *I*_R_, (f) electron transfer number (*n*) & %HO_2_^–^ yield, and (g) EIS spectra of the as-synthesized SnFe/SNC_*T* (where *T* = 750, 850, and 950 °C) and commercial 20% Pt/C; (h) ORR polariztion curves and (i) ECSA *C*_dl_ values of SnFe/SNC_850, Sn/SNC_850, Fe/SNC_850, and Fe/NC_850.

Linear Sweep Voltammetry (LSV) curves (Figure 6b), recorded at 1600 rpm, provided further insights into ORR performance using key parameters: onset potential (*E*_onset_), half-wave potential (*E*_1/2_), and limiting current density (*J*_L_).^57^ SnFe/SNC_850 demonstrated an impressive *E*_onset_ of 1.04 V and an *E*_1/2_ of 0.86 V vs RHE. These values significantly surpass those of commercial 20% Pt/C (*E*_onset_: 0.98 V, *E*_1/2_: 0.82 V) and the other synthesized catalysts, SnFe/SNC_750 (*E*_onset_: 0.94 V, *E*_1/2_: 0.77 V) and SnFe/SNC_950 (E_onset_: 0.95 V, *E*_1/2_: 0.79 V). A more positive *E*_1/2_ indicates higher ORR activity, as it signifies the catalyst’s ability to achieve substantial current at a lower overpotential, a critical factor for efficient fuel cell operation.^13^ The enhanced performance of SnFe/SNC_850 (synthesized at 850 °C) can be attributed to several factors: (i) increased graphitization of the carbon support (consistent with XRD and Raman data), (ii) the formation of well-defined bamboo-like tubular networks that facilitate electron transport and mass transfer, and (iii) an enhanced surface area. Additionally, EDS analysis confirmed higher N and S atomic percentages in SnFe/SNC_850, suggesting a greater abundance of M-N_*x*_ and M-S_*x*_ active sites, corroborating the XPS analysis. Conversely, the slight performance decrease observed for SnFe/SNC_950 may be due to Sn evaporation at the higher temperature (supported by XRD and EDS), loss of nanoparticle-confining bamboo nodes, and degradation of the tubular network structure (evident in SEM images). The confinement of nanoparticles within bamboo cavities and the integrity of the tubular network are crucial for optimizing conductivity, modulating the electronic environment of active sites (Fe–N_*x*_ and Fe–S_*x*_), and ensuring efficient mass transport.^58^

Superior ORR kinetics for SnFe/SNC_850 are further confirmed by its smaller Tafel slope of 80.1 mV dec^–1^ (Figure 6c). This kinetic advantage correlates with a larger electrochemically active surface area (ECSA), as indicated by its higher double-layer capacitance (*C*_dl_) of 4.1 mF cm^–2^ (Figures 6d and S5­(a–d) of the Supporting Information). A larger ECSA implies more accessible active sites for ORR,^59^ which is also consistent with its higher normalized current density (JECSA) (Figure S5e and Table S4 of Supporting Information) and its high mass activity (Figure S6 of Supporting Information). Moreover, SnFe/SNC_850 achieved a *J*_L_ of 5.31 mA cm^–2^, closely approaching the theoretical value for a 4-electron ORR pathway in O_2_-saturated 0.1 M KOH.

The electron transfer number (n) and hydrogen peroxide (HO_2_^–^) yield were determined from RRDE measurements to elucidate the ORR pathway selectivity. RRDE results (Figure 6e) show that SnFe/SNC_850 exhibited high disk currents (*J*_D_) and low ring currents (*I*_R_), a behavior comparable to that of 20% Pt/C. This indicates efficient O_2_ reduction primarily to OH^–^ at the disk, with minimal formation of the HO_2_^–^ intermediate, resulting in a low HO_2_^–^ yield. Quantitatively, over the potential range of 0.2 to 0.8 V vs RHE (Figure 6f), SnFe/SNC_850 demonstrated a remarkably low HO_2_^–^ yield (ranging from 0.34% to 0.038%) and an electron transfer number (*n*) consistently near 4 (3.99 to 4.00). This performance is superior to SnFe/SNC_750 (%HO_2_^–^: 1.7–0.26%, *n*: 3.96–3.99) and SnFe/SNC_950 (%HO_2_^–^: 4.47–0.3%, *n*: 3.91–3.99), and closely rivals that of 20% Pt/C (%HO_2_^–^: 0.12–0.06%, *n*: 3.998–4.00). These findings confirm the high selectivity of SnFe/SNC_850 for the direct 4-electron ORR pathway, resulting in high efficiency. The negligible HO_2_^–^ yield is particularly significant, as it suggests that SnFe/SNC_850 inhibits detrimental Fenton reactions.^58^ Furthermore, electrochemical impedance spectroscopy (EIS) analysis (Figure 6g and Table S5 of the Supporting Information) reveals that the synthesized SnFe/SNC_850 catalyst exhibits the lowest charge-transfer resistance (*R*_ct_) of 9.314 Ω, which is significantly lower than that of commercial 20% Pt/C (11.75 Ω) and the other synthesized catalysts. This reduced *R*_ct_ indicates faster interfacial electron-transfer kinetics and improved charge-transport characteristics under ORR conditions, contributing to the superior catalytic activity of SnFe/SNC_850.

To elucidate the synergistic effect between tin (Sn) and iron (Fe) on ORR activity, a series of control catalysts, Fe/NC_850, Fe/SNC_850, and Sn/SNC_850 (all synthesized at 850 °C), were prepared, and their performance was benchmarked against the bimetallic SnFe/SNC_850. As shown by the half-wave potentials (*E*_1/2_) in Figure 6h, sulfur doping significantly enhanced the activity of the iron-based catalyst: Fe/SNC_850 (0.80 V vs RHE) exhibited a more positive *E*_1/2_ than Fe/NC_850 (0.71 V). This improvement is likely attributed to the formation of M-S_*x*_ configurations and sulfur vacancies, which can act as active sites.^4^ Interestingly, the Sn/SNC_850 catalyst (*E*_1/2_ = 0.81 V) showed slightly higher activity than Fe/SNC_850, suggesting Sn’s intrinsic ORR catalytic capability, even in the S-doped carbon matrix. Crucially, the bimetallic SnFe/SNC_850 catalyst achieved the most positive *E*_1/2_ of 0.86 V, significantly outperforming all single-metal counterparts. This pronounced enhancement strongly indicates a synergistic catalytic effect arising from the copresence of Sn and Fe. This synergistic improvement in activity is further corroborated by the electrochemical surface area (ECSA) calculations (Figures 6i and S7 of Supporting Information).

X-ray Photoelectron Spectroscopy (XPS) analysis of SnFe/SNC_850 identified the presence of Fe–N_*x*_ and Fe–S_*x*_ moieties, widely recognized as primary ORR active sites.^5^ The Fe K-edge EXAFS analysis further confirms the existence of Fe–N_*x*_ and Fe–S_*x*_ coordinations. Complementarily, X-ray Diffraction (XRD) patterns revealed Fe_3_SnC and FeS phases. These intermetallic and sulfide phases are proposed to play a crucial secondary, yet synergistic, role by enhancing electronic conductivity and stabilizing the Fe-based active sites. Notably, high-resolution XPS spectra showed no evidence of Sn–N_*x*_ or Sn–S_*x*_ coordination. This suggests that Sn species may not act as catalytic centers but rather modulate the electronic environment of adjacent Fe active sites, thereby enhancing activity. Therefore, the superior ORR activity of SnFe/SNC_850 is attributed to a synergistic interplay: the primary catalysis occurs at atomically dispersed Fe–N_*x*_/Fe–S_*x*_ sites. At the same time, the Fe–Sn intermetallic and FeS sulfide domains provide multifunctional support by optimizing electronic properties and stabilizing the Fe–N_*x*_/Fe–S_*x*_ active centers.^60^

The durability of SnFe/SNC_850 was evaluated via an accelerated deterioration test (ADT). This involved subjecting the catalyst to 30,000 continuous cyclic voltammetry (CV) cycles under N_2_-saturated conditions, followed by linear sweep voltammetry (LSV) measurements under O_2_-saturated conditions to compare initial and final ORR activities. As depicted in Figure 7a, SnFe/SNC_850 demonstrated excellent stability, with its half-wave potential (*E*_1/2_) decaying by only 20 mV (vs RHE). This is significantly less than the 50 mV decay observed for commercial 20% Pt/C under identical conditions (Figure S8 of the Supporting Information). After completing the 30,000-cycle ADT stability test, a comprehensive poststructural analysis of SnFe/SNC_850 was carried out using XRD, SEM, and TEM. The SEM image (Figure S9a of Supporting Information) and the EDS (inset, Figure S9a of Supporting Information) confirm that the SnFe/SNC_850 retains its bamboo-like tubular morphology and chemical compositions. The dark spots in the TEM (Figure S9b of the Supporting Information) and the *d*-spacings in the HRTEM (Figure S9c,d of Supporting Information) reveal the presence of Fe_3_SnC intermetallic encapsulated in the carbon, indicating that no significant dissolution of the nanoparticles occurred during the stability test. The XRD patterns (Figure S9e of the Supporting Information) further support this, revealing the different diffraction planes corresponding to the Fe_3_SnC phase. This observation is further supported by the preserved tubular morphology in SEM/TEM and unchanged Fe_3_SnC peaks in XRD. Additionally, to evaluate structural robustness and elemental retention, ICP-OES analysis was performed on SnFe/SNC_850 before and after 30,00 cycles of ADT durability testing. As shown in Table S6 of Supporting Information, the Fe and Sn contents remained nearly unchanged (Fe: 7.42 wt % → 7.38 wt %; Sn: 3.56 wt % → 3.53 wt %), indicating negligible leaching. These results demonstrate that the SnFe/SNC_850 catalyst retained its structure and composition during the extended stability test, which correlates strongly with its stable, selective performance.

**Figure 7:**
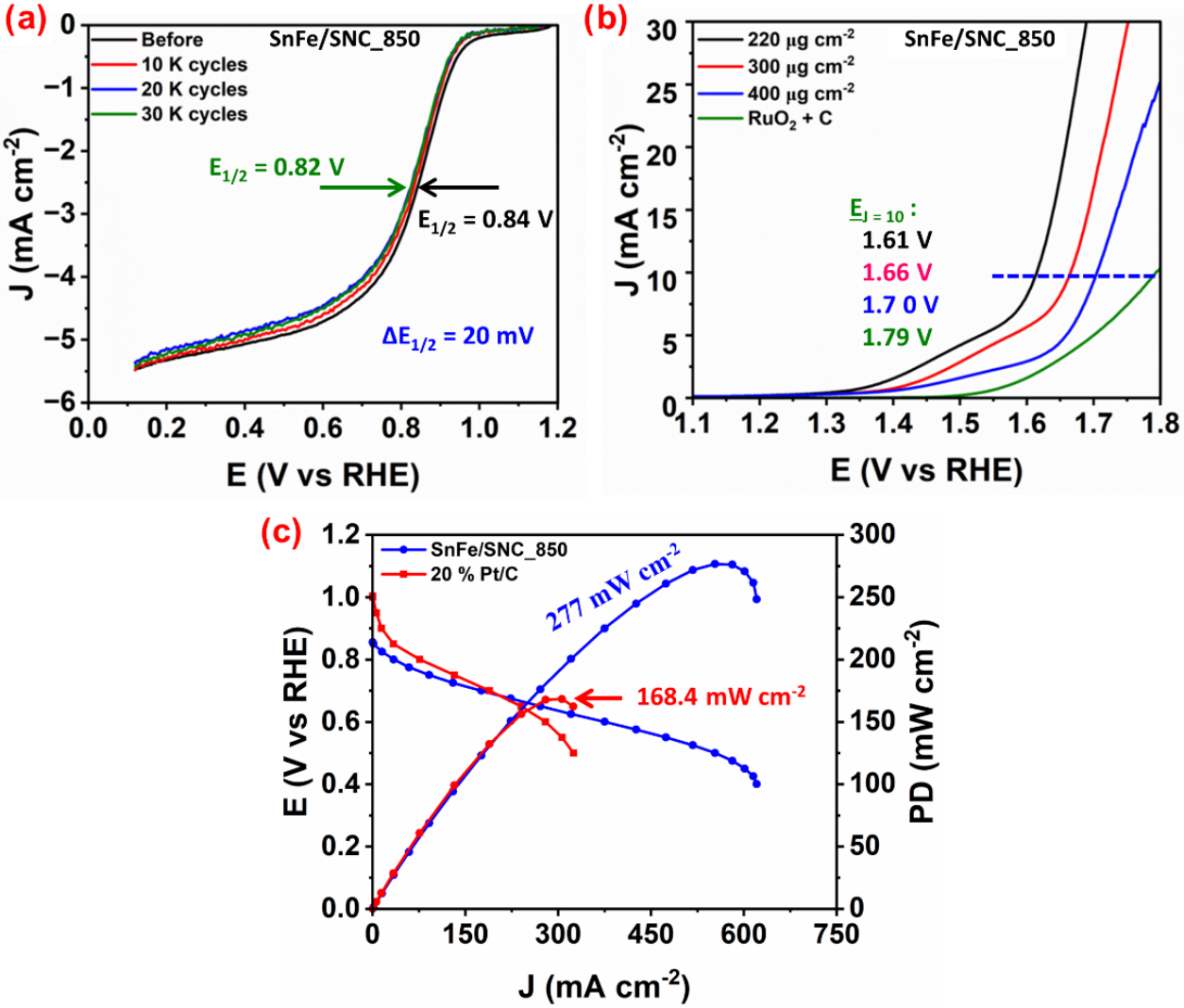
(a) Stability after 30,000 cycles, (b) OER LSV curves, and (c) AEMFC cathodes using SnFe/SNC_850 and 20% Pt/C.

Beyond its notable ORR performance, a catalyst’s OER activity is crucial for establishing its bifunctional capabilities. Interestingly, the beneficial roles of metal and sulfur species in enhancing ORR activity extend to the OER. Specifically, sulfur doping and the synergistic presence of Sn and Fe also boost the OER catalytic activity of SnFe/SNC_850.^5,20^ It is proposed that Sn promotes OER by facilitating OH^–^ ligand coordination to the transition-metal center and weakening the M–OH^–^ bond, thereby lowering the energy barrier to the OER process.^61^ Consequently, the OER catalytic activity of SnFe/SNC_850 was investigated at various loadings using a rotating disk electrode (RDE) in a three-electrode setup with 1 M KOH electrolyte. Its performance was benchmarked against commercial RuO_2_ + C (2:8 ratio). OER activity was quantified by the potential required to achieve a current density of 10 mA cm^–2^ (denoted as *E*_*J*=10_), where a lower *E*_*J*=10_ value indicates superior catalytic performance. Figure 7b illustrates the OER performance of SnFe/SNC_850 at different loadings relative to the RuO_2_ + C benchmark. Notably, SnFe/SNC_850 at a 200 μg cm^–2^ loading exhibited the lowest *E*_*J*=10_ of 1.61 V (vs RHE). This corresponds to a remarkably low overpotential of 340 mV, signifying its excellent OER catalytic performance, particularly at this optimized lower loading. Furthermore, the SnFe/SNC_850 at this optimized lower loading exhibited a remarkably low potential gap (Δ*E* = *E_J_*_=10_ – *E*_1/2_ = 1.61 V – 0.86 V = 750 mV), signifying its superior bifunctional ORR/OER catalytic performance. This excellent OER catalytic performance of the synthesized SnFe/SNC_850 at the loading of 200 μg cm^–2^ correlates with its larger electrochemically active surface area (ECSA), as indicated by its higher double-layer capacitance (*C*_dl_) of 7.68 mF cm^–2^ (Figure S10­(a–e) of the Supporting Information) and its larger ECSA normalized current density *J*_ECSA_ of 0.043 mA cm^–2^ (Figure S10f and Table S7 of the Supporting Information). These superior bifunctional performances arise from the synergistic contributions of Fe–N_*x*_/Fe–S_*x*_ moieties and Fe_3_SnC/FeS intermetallic domains, each playing distinct roles in the ORR and OER pathways. The Fe–N*_x_* sites serve as the primary centers for O_2_ adsorption and reduction, efficiently stabilizing *OOH and facilitating O–O bond cleavage, as supported by previous DFT studies showing optimal *OOH and *OH binding energies on Fe–N_*x*_ moieties.^62,63^ Fe–S_*x*_ sites modulate the electronic structure of Fe centers, enhancing intermediate stabilization and contributing to bifunctionality.^64^ Meanwhile, Fe_3_SnC and FeS domains act as robust OER centers by promoting *OH and *O formation, with Sn incorporation further tuning the adsorption energies and facilitating oxygen evolution.^65^ The interfaces between these domains and the N, S-codoped carbon matrix generate unique active environments that favor the intermediate transformations in ORR and OER through interfacial electronic coupling.^4^ Furthermore, the Fe K-edge XANES reveals that Fe in SnFe/SNC_850 exhibits an intermediate oxidation state between Fe^0^ and Fe^2+^, consistent with partially electron-enriched Fe centers capable of facilitating O_2_ activation. The corresponding EXAFS spectra further confirm the coexistence of Fe–N_*x*_ (≈1.49 Å) and Fe–S_*x*_ (≈1.79 Å) coordination environments, along with a weaker Fe–Fe scattering peak (∼2.4 Å) associated with Sn–Fe intermetallic clusters. This mixed coordination structure provides complementary functionalities: the Fe–N_*x*_/Fe–S_*x*_ moieties modulate the adsorption energies of *OOH/*OH and serve as highly active ORR sites, while the Sn–Fe domains enhance electronic conductivity and stabilize *O/*OOH intermediates relevant to OER.^1,66,67^ Such dual-site cooperation results in accelerated reaction kinetics and higher intrinsic turnover frequencies,^1^ which is consistent with the catalyst’s ECSA-normalized ORR activity and its higher half-wave potential (0.86 V vs RHE). These mechanistic insights collectively demonstrate that the enhanced performance of SnFe/SNC_850 arises from synergistic electronic modulation rather than solely from an increased electrochemical surface area.

Chronoamperometry was used to assess the catalyst’s OER stability. Figure S11 of the Supporting Information shows that the SnFe/SNC_850 exhibited a current decay of only 2% after 24h, showing superior durability than the commercial RuO_2_, which exhibited a current decay of 19% after 24h. Furthermore, the ICP-OES analysis performed on SnFe/SNC_850 before and after the 24h chronoamperometric durability test showed the Fe and Sn contents remained nearly unchanged (Fe: 7.42 wt % → 7.41 wt %; Sn: 3.56 wt % → 3.55 wt %), indicating negligible leaching (Table S6 of the Supporting Information).

The practical applicability and excellent ORR activity of SnFe/SNC_850 were further validated through single-cell tests in an actual anion-exchange membrane fuel cell (AEMFC) platform, as shown in Figure 7c. SnFe/SNC_850 achieved a maximum peak power density of 277 mW cm^–2^, outperforming commercial 20% Pt/C (168.4 mW cm^–2^) by 40%. Further, the electrochemical durability of SnFe/SNC_850 was evaluated through single-cell AEMFC testing. Constant-current operation was conducted at 0.3 A cm^–2^ and 65 °C under H_2_/O_2_ (150 kPa­(abs)) using a Sustainion X37–50 RT membrane, followed by load-cycling durability between 0.2 ↔ 0.8 A cm^–2^ with a 60 s dwell for 1000 cycles under identical conditions. As shown in Figure S12 of Supporting Information, both the polarization (E–J) curves and voltage–time profiles reveal that SnFe/SNC_850 maintained a nearly constant voltage (∼0.72 V) during 20 h of operation and retained ∼96% of its initial performance after 1000 load cycles, markedly surpassing 20 wt % Pt/C (∼89% retention). The minimal voltage decay demonstrates the robust interfacial contact and structural integrity of the SnFe/SNC_850 catalyst under operating conditions.

Furthermore, comparison with various nonprecious dual-metal catalysts reported in the literature (Table S8 of the Supporting Information) indicates that the as-synthesized SnFe/SNC_850 exhibits competitive half-cell ORR activity and delivers a superior AEMFC power density (277 mW cm^–2^), highlighting its strong practical catalytic performance.^3,5,10,13,15,16,20,21,23,45,68^ These comprehensive results underscore the significant potential of the synthesized SnFe/SNC_850 catalyst for practical fuel cell applications.

Figure 8 shows (a,b) the total DOS, (c,d) the OER free-energy diagrams at *U* = 0 and 1.23 V, and (e–j) the atomic models of undoped and doped g-C_3_N_4_. As shown in Figure 8(a,b), pristine g-C_3_N_4_ exhibits a wide band gap, consistent with its intrinsic semiconducting nature. Single doping with Fe or Sn narrows the band gap and introduces states near the Fermi level. Co-doping with Fe and Sn, or Fe and S, further enhances the metallic character of the system. The triple-doped (Fe, Sn, S) structure displays the highest density of states at the Fermi level, indicating significantly improved electrical conductivity, which is beneficial for electrocatalytic applications. In Figure 8c, at *U* = 0 V, all models show an uphill step for *OH adsorption, followed by exothermic deprotonation to *O, evidencing relatively strong stabilization of atomic oxygen intermediates. Fe doping provides the strongest binding to oxygen-containing species, while the incorporation of Sn substantially weakens the adsorption energies of all intermediates. Additional S codoping partially modulates this effect, yielding intermediate binding strengths. At *U* = 1.23 V (Figure 8d), the O_2_ release step remains the potential-determining step for most models. The Fe–Sn codoped system exhibits the smallest thermodynamic barrier among the investigated structures, demonstrating that Sn effectively tunes the adsorption energies toward a more balanced regime. The further introduction of S into the triple-doped system enhances electronic conductivity while maintaining a favorable intermediate binding state, consistent with the experimentally observed synergistic electronic modulation. These results highlight the critical role of Sn in optimizing oxygen-intermediate adsorption and the complementary contribution of S to improving charge transport. These results indicate that Fe provides strong binding, while Sn tunes the adsorption energy. S codoping further refines the intermediate binding strength via electronic modulation, resulting in a well-balanced adsorption configuration. Even though the free-energy analysis was performed for the OER pathway, the electronic structure modulation induced by Fe, Sn, and S codoping is expected to influence both ORR and OER kinetics as both reactions involve adsorption and activation of oxygen-containing intermediates at the same catalytic sites, which are governed by the electronic structure of the active centers.^69^ These insights, akin to multisite interactions in high-entropy materials^70^ that modulate affinities for intermediates, highlight the role of dopant interplay in enhancing catalytic performance for practical AEMFC applications. This mechanistic insight provides valuable guidance for further compositional optimization of nonprecious catalysts.

**Figure 8:**
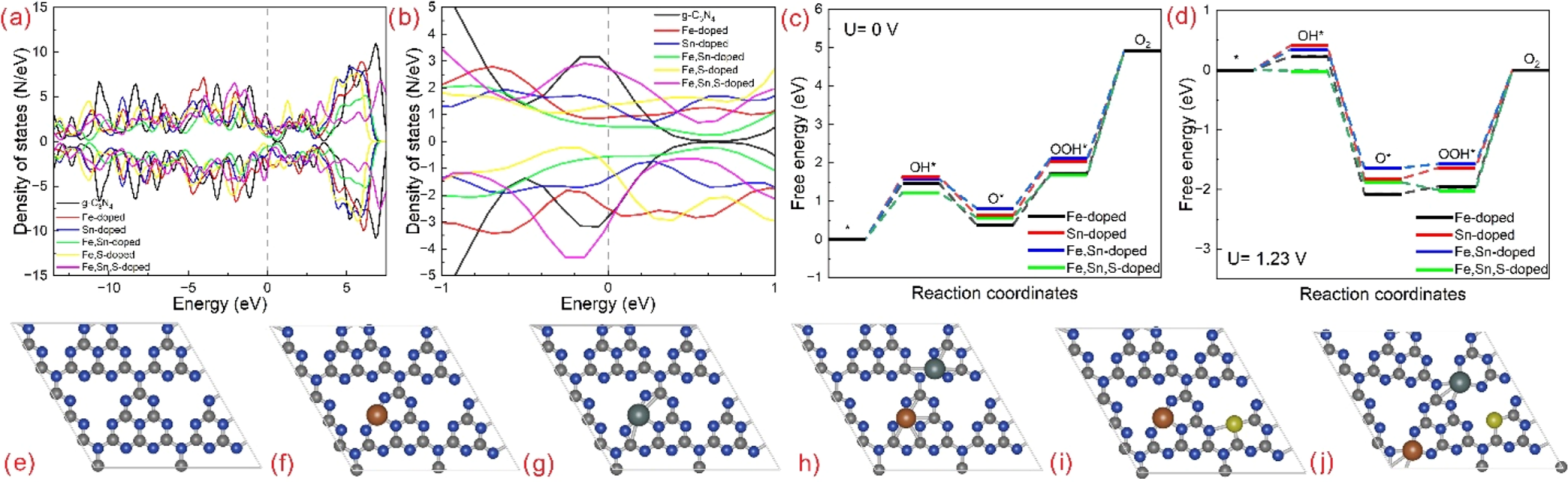
(a) Total DOS of undoped, Fe-doped, Sn-doped, (Fe, Sn)-doped, (Fe, S)-doped, (Fe–Sn, S)-doped g-C_3_N_4_, (b) Total DOS at the Fermi level, (c) Standard free energy diagram of the OER process at 0 V on the different samples, (d) Standard free energy diagram of the OER process at 1.23 V, and Models of (e) undoped g-C_3_N_4_, (f) Fe-doped g-C_3_N_4_, (g) Sn-doped g-C_3_N_4_, (h) Fe, Sn-doped g-C_3_N_4_, (i) Fe, S-doped g-C_3_N_4_, and (j) Fe, Sn, S-doped g-C_3_N_4_. Atoms are marked as follows: N (blue), C (gray), Fe (brown), Sn (teal), and S (yellow).

## 4 Conclusions

This study reports a facile synthesis of S, N codoped bamboo-like tubular carbon structures anchoring bimetallic Sn–Fe nanoparticles, which exhibit enhanced bifunctional ORR and OER catalytic activity. Scanning Electron Microscopy (SEM) confirmed the formation of a distinct bamboo-like tubular morphology upon pyrolysis at 850 °C. Transmission Electron Microscopy (TEM) analysis revealed that the nanoparticles are predominantly confined within the nodes of these bamboo-like cavities. These bamboo-like carbon structures possess a specific surface area of 76.9 m^2^ g^–1^ and abundant porosity, facilitating electrolyte transport and enhancing overall ORR/OER catalysis. SnFe/SNC_850 exhibits excellent ORR performance and remarkable stability, showing only a negligible decay in its half-wave potential (*E*_1/2_) after 30,000 accelerated aging cycles. The bifunctionality of the as-synthesized SnFe/SNC_850 was further evidenced by its superior OER catalytic activity, requiring an overpotential of only 340 mV to achieve a current density of 10 mA cm^–2^. An Alkaline Exchange Membrane Fuel Cell (AEMFC) employing SnFe/SNC_850 as the catalyst achieved a high maximum peak power density of 277 mW cm^–2^. The superior performance of this dual-metallic catalyst is attributed to synergistic interactions between Fe–N_*x*_ and Fe–S_*x*_ moieties coupled with the presence of intermetallic Fe_3_SnC and FeS phases. Furthermore, forming well-defined bamboo-like tubular networks at 850 °C promotes efficient mass transfer, enhancing catalytic performance. Collectively, these findings highlight SnFe/SNC_850 as a highly promising bifunctional electrocatalyst for ORR and OER applications.

## Supplementary Material



## Notes

Additional experimental data (figures and tables), including polarization curves of NC, SNC and SnFe/SNC_850, SEM images and EDS survey of synthesized catalysts, XPS survey spectrum, CV curves at different scan rates for *C*_dl_ determination (ORR), ECSA normalized polarization curves (ORR), mass activity, post-ADT morphological and structural characterizations, chronoamperometric curves, *C*_dl_ determination and ECSA normalized polarization curves (OER), polarization curves of single-cell durability, and Tables of SEM-EDS elemental composition, percent concentration of oxidations states and bond configurations determined from high resolution XPS spectra, ECSA normalized current densities (ORR), EIS fitting results, pre-ADT and post-ADT ICP-OES results, ECSA normalized current densities (OER), and performance comparison of synthesized catalyst (PDF)
