# Supplementary material for: Sn–Fe Dual-Metallic Nanoparticles on S,N-Codoped g‑C3N4‑Derived Tubular Carbon as an Efficient Bifunctional Catalyst for Oxygen Reduction Reaction and Oxygen Evolution Reaction
Source: ACS Appl Mater Interfaces. 2026 Apr 25;18(17):24456–70. doi: 10.1021/acsami.6c00103 (PMC13154135; doi:10.1021/acsami.6c00103)
Supplement: Supplementary file 1 [file am6c00103_si_001.pdf]

Supporting Information

Sn-Fe Dual-Metallic Nanoparticles on S,N-codoped g-C_3N_4-derived Tubular Carbon as an Efficient Bifunctional Catalyst for Oxygen Reduction Reaction and Oxygen Evolution Reaction

Berhanu Telay Mekonnen,[1] Daniel Manaye Kabtamu,[1] Sun-Tang Chang,[1] Guan-Cheng Chen,[1] Amil Aligayev,[2] Francisco Javier Dominguez-Gutierrez,[2] Yao-Ming Wang,[3] Sheng-Yu Wang,[3] Wenyi Huo,[2]* Chen-Hao Wang[1,4,5]*

[1.] Department of Materials Science and Engineering, National Taiwan University of Science and Technology, Taipei 106335, Taiwan

[2.] NOMATEN Centre of Excellence, National Centre for Nuclear Research, Otwock 05-400, Poland

[3.] Maritime Innovation & Industry Promotion Department, Metal Industries Research & Development Centre, Kaohsiung 811160, Taiwan

[4.] Advanced Manufacturing Research Center, National Taiwan University of Science and Technology, Taipei 106335, Taiwan

[5.] Research Center for Critical Issues, Academia Sinica, Tainan 711010, Taiwan

*Corresponding authors:

Chen-Hao Wang: Email: chwang@mail.ntust.edu.tw. Tel: 886-2-2730-3715, Fax: 886-2-2737-6544.

Wenyi Huo: Email: wenyi.huo@ncbj.gov.pl.

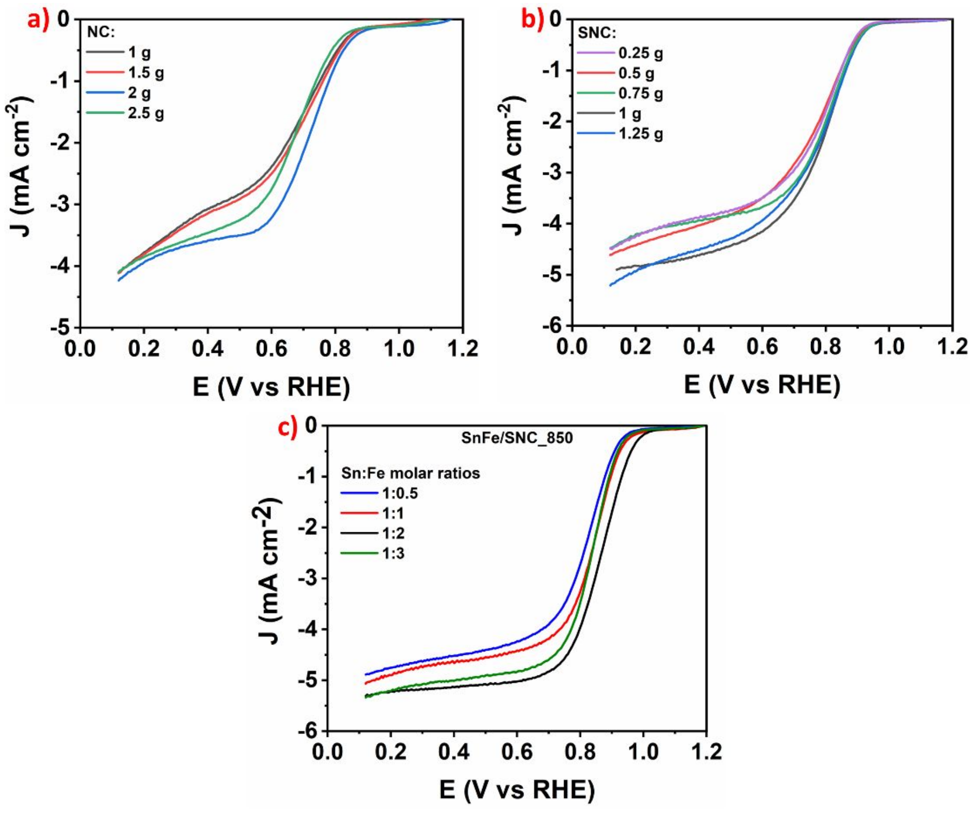

Figure S1. ORR polarization curves of (a) N-doped carbons (NC) prepared with different g-C_3N_4 loadings (1.0, 1.5, 2.0, and 2.5 g), (b) S,N-co-doped carbons (SNC) obtained with varying thiourea amounts (0.25, 0.5, 0.75, 1.0, and 1.25 g), and (c) SnFe/SNC_850 catalysts synthesized with different Sn:Fe molar ratios. Electrochemical measurements were conducted in O_2-saturated 0.1 M KOH at room temperature using RRDE (rotation rate: 1600 rpm; scan rate: 10 mV s^{-1}; catalyst loading: 200 μg cm^{-2}). The NC sample prepared with 2 g of g-C_3N_4 and the SNC sample synthesized with 1 g of thiourea exhibited the most positive onset potentials and the highest diffusion-limited current densities. It was therefore selected as the optimized S,N-doped carbon frameworks for the synthesis of SnFe/SNC_T catalysts. Among the investigated metal compositions, SnFe/SNC_850 with a Sn:Fe molar ratio of 1:2 displayed the most positive onset potential and the highest diffusion-limited current density, and was thus chosen as the optimized metal loading.

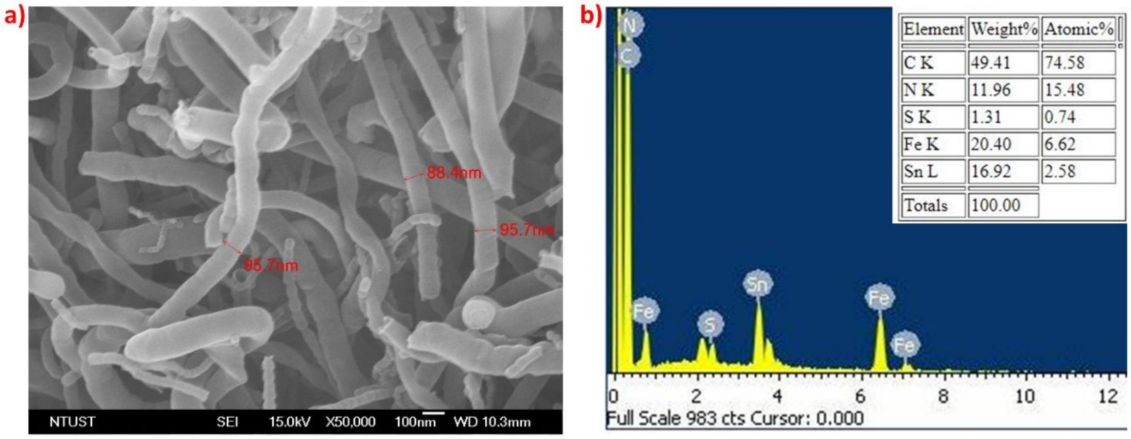

Figure S2. (a) SEM image of SnFe/SNC_850 depicting its tube diameter, and (b) SEM-EDS survey spectrum of SnFe/SNC_850.

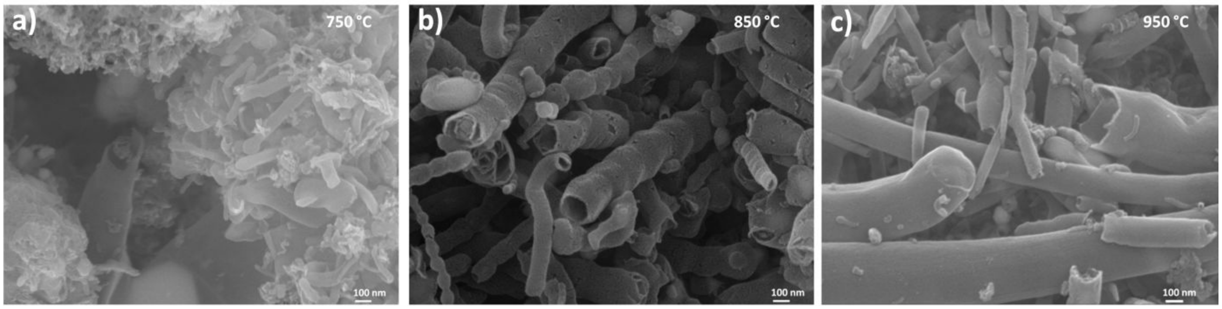

Figure S3. The effect of pyrolysis temperature on the morphology of (a) SnFe/SNC_750, (b) SnFe/SNC_850, and (c) SnFe/SNC_950.

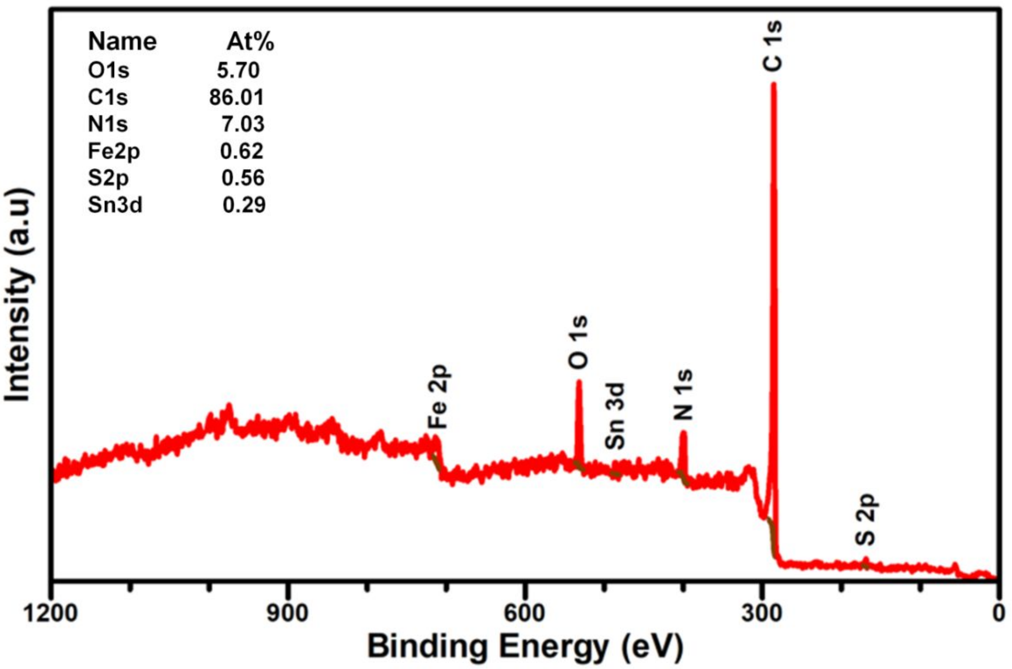

Figure S4. XPS survey spectrum of SnFe/SNC_850.

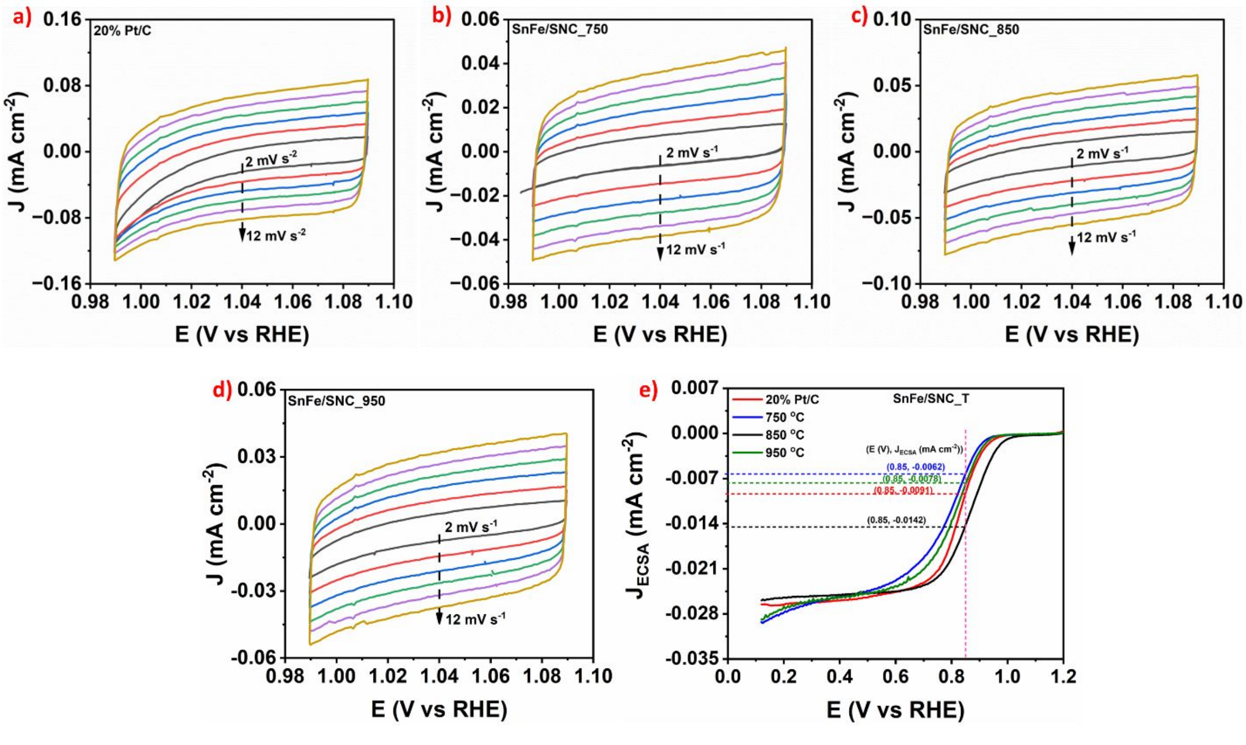

Figure S5. CV curves (a) 20% Pt/C, (b) SnFe/SNC_750, (c) SnFe/SNC_850, and (d) SnFe/SNC_950 at various scan rates in 0.1 M KOH in a potential window of non-Faradic current,

and (e) ECSA normalized ORR polarization curves of SnFe/SNC_750, SnFe/SNC_850, and SnFe/SNC_950.

Calculation of electrochemically active surface area (ECSA)

Cyclic voltammetry (CV) of the catalyst was carried out in a potential range of 0.99 to 1.09 V (*vs*. RHE, in the non-Faradaic current region) at the scan rates of 2, 4, 6, 8, 10, and 12 mV s^{-1}. Then, a linear plot was drawn between the capacitive current densities at 1.04 V ($\frac{\Delta J}{2} = \frac{J_a - J_c}{2}$) and the scan rate. The fitted slope of the plot corresponds to the electrical double-layer capacitance (Cdl), which can be used to estimate the electrochemically active surface area (ECSA). The ECSA was calculated from the C_{dl} according to the equation:

$$ECSA=\frac{C_{dl}}{C_s} \quad (1)$$

where C_s is the specific capacitance (20 μF cm^{-2} for the synthesized catalyst and 30 μF cm^{-2} for 20 % Pt/C in 0.1M KOH).

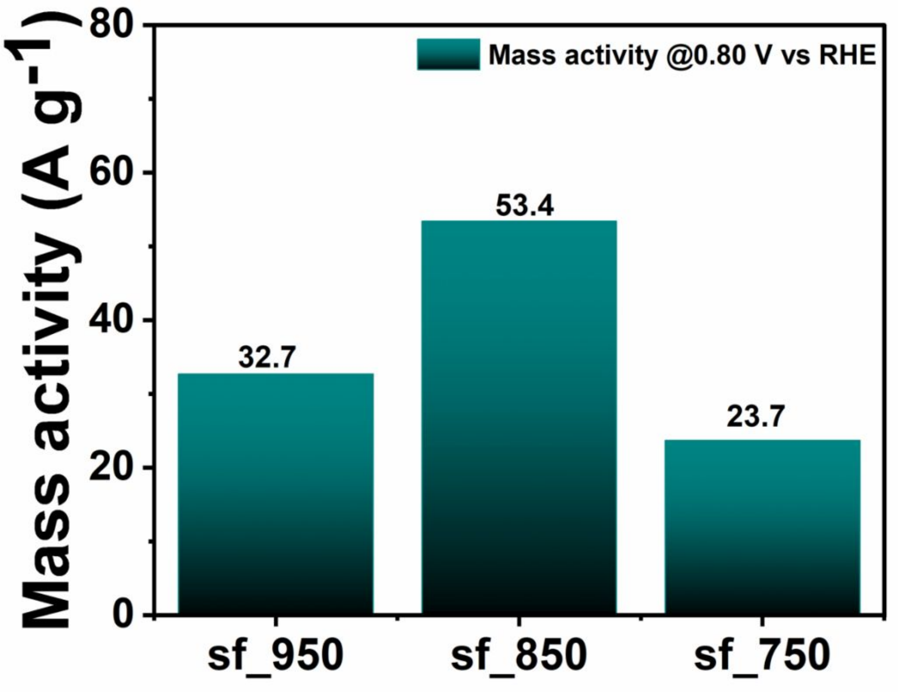

Figure S6. Mass activity of the as-synthesized catalysts calculated at 0.80 V [where sf_950, sf_850, & sf_750 = SnFe/SNC_950, SnFe/SNC_850, & SnFe/SNC_750].

Mass activity calculation:

The mass activity calculation for the as-synthesized catalysts is performed by dividing the current density (J) at 0.80 V by the metal loading of each catalyst. The metal loading is determined from TEM-EDS atom weight % and the catalyst loading on the working electrode.

$$\boldsymbol{Mass\ Activity = \frac{Current\ density\ at\ 0.80\ V\ (J_{@0.80V})}{Metal\ loading}}$$

$$\boldsymbol{Metal\ loading = atom\ weight\ \%\ x\ catalyst\ loading}$$

$$\boldsymbol{Mass\ activity = \frac{0.00395\ A\ cm^{-2}}{0.37\ x\ 0.0002\ g\ cm^{-2}} = 53.4\ A\ g^{-1}}$$

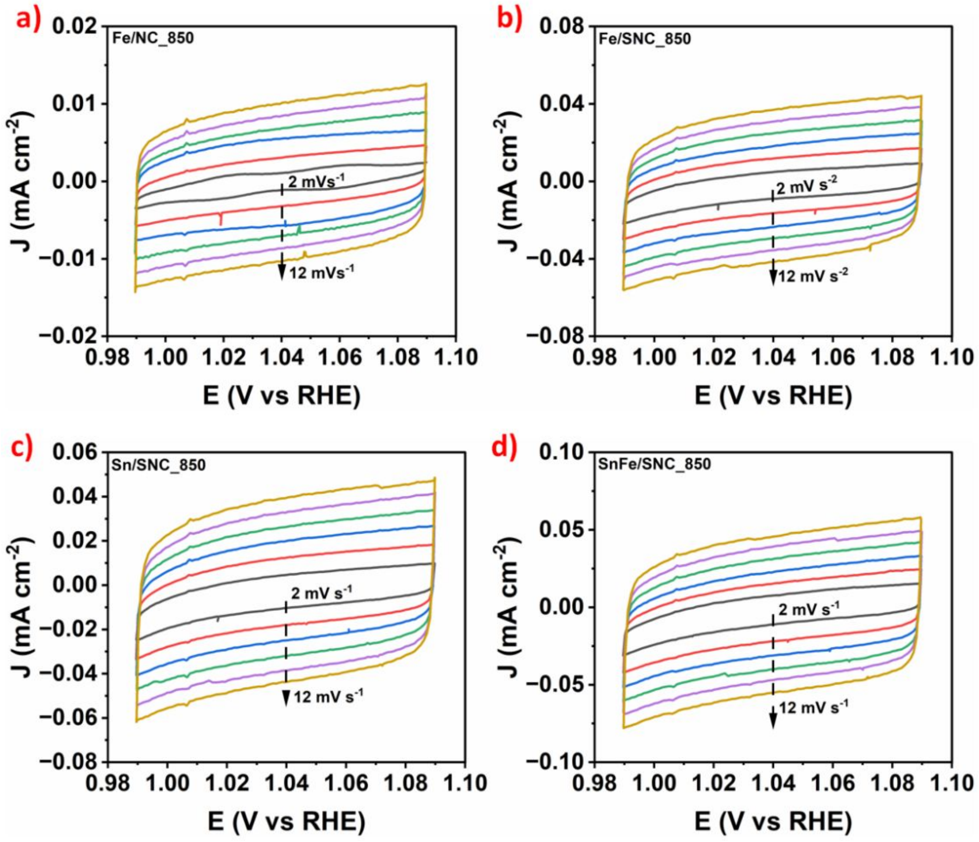

Figure S7. CV curves (a) Fe/SNC_850, (b) Fe/NC_850, (c) Sn/SNC_850, and (d) SnFe/SNC_850 at various scan rates in 0.1 M KOH in a potential window of non-Faradic current.

N.B.: The CV-based determination of C_{dl} in the non-Faradaic region is presented in **Figure S5**.

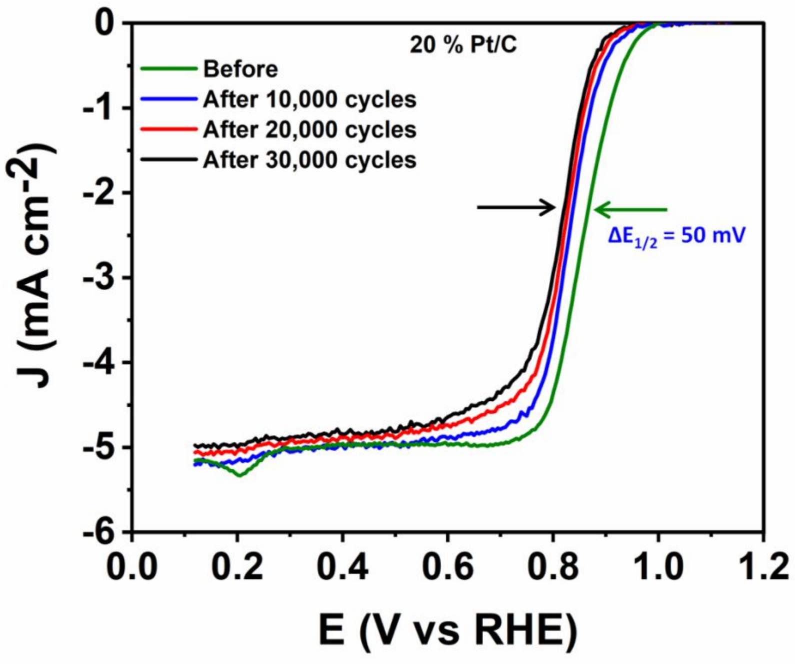

Figure S8. Stability of 20% Pt/C for 30,000 cycles.

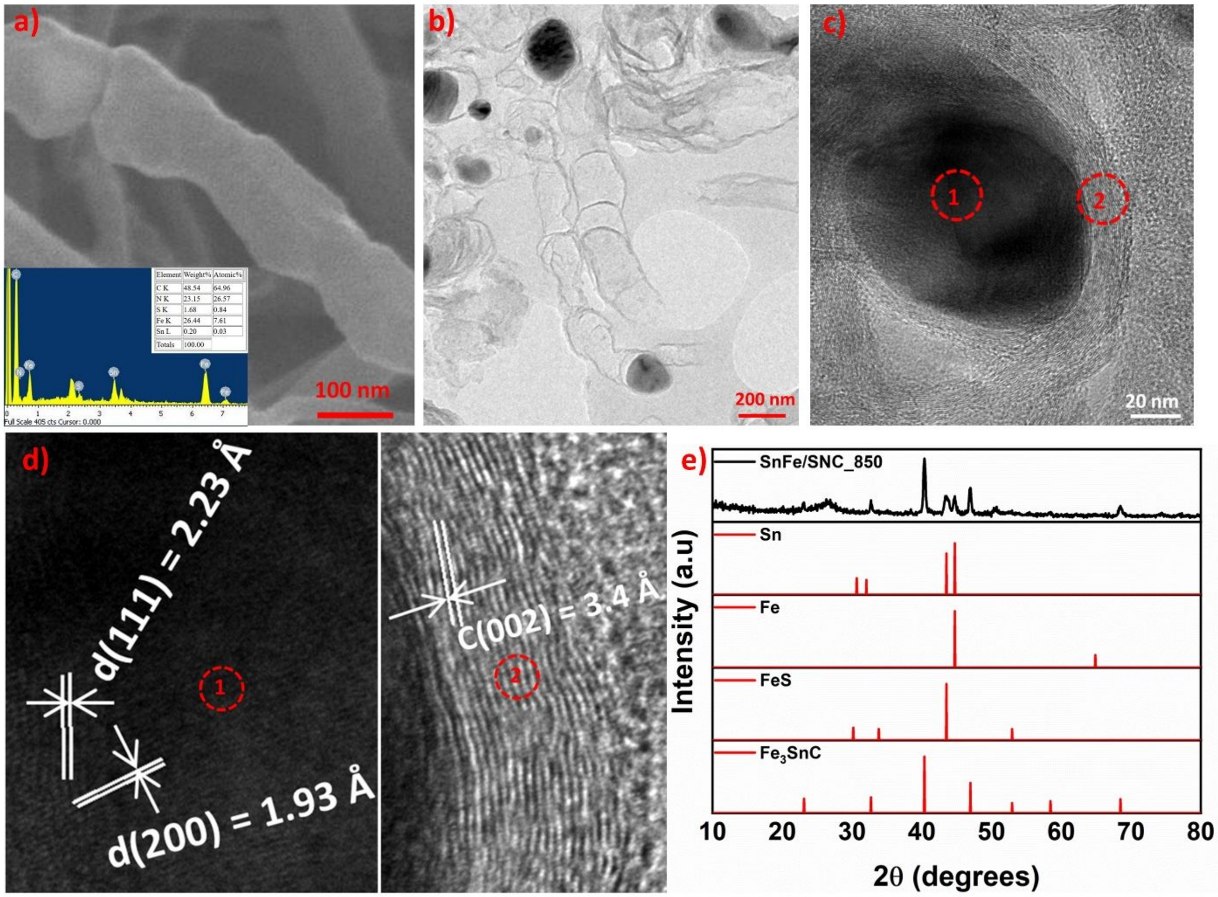

Figure S9. (a) SEM, (b) TEM, (c,d) HRTEM images, and (e) XRD spectrum of SnFe/SNC_850 taken after 30,000 cycles ADT stability test.

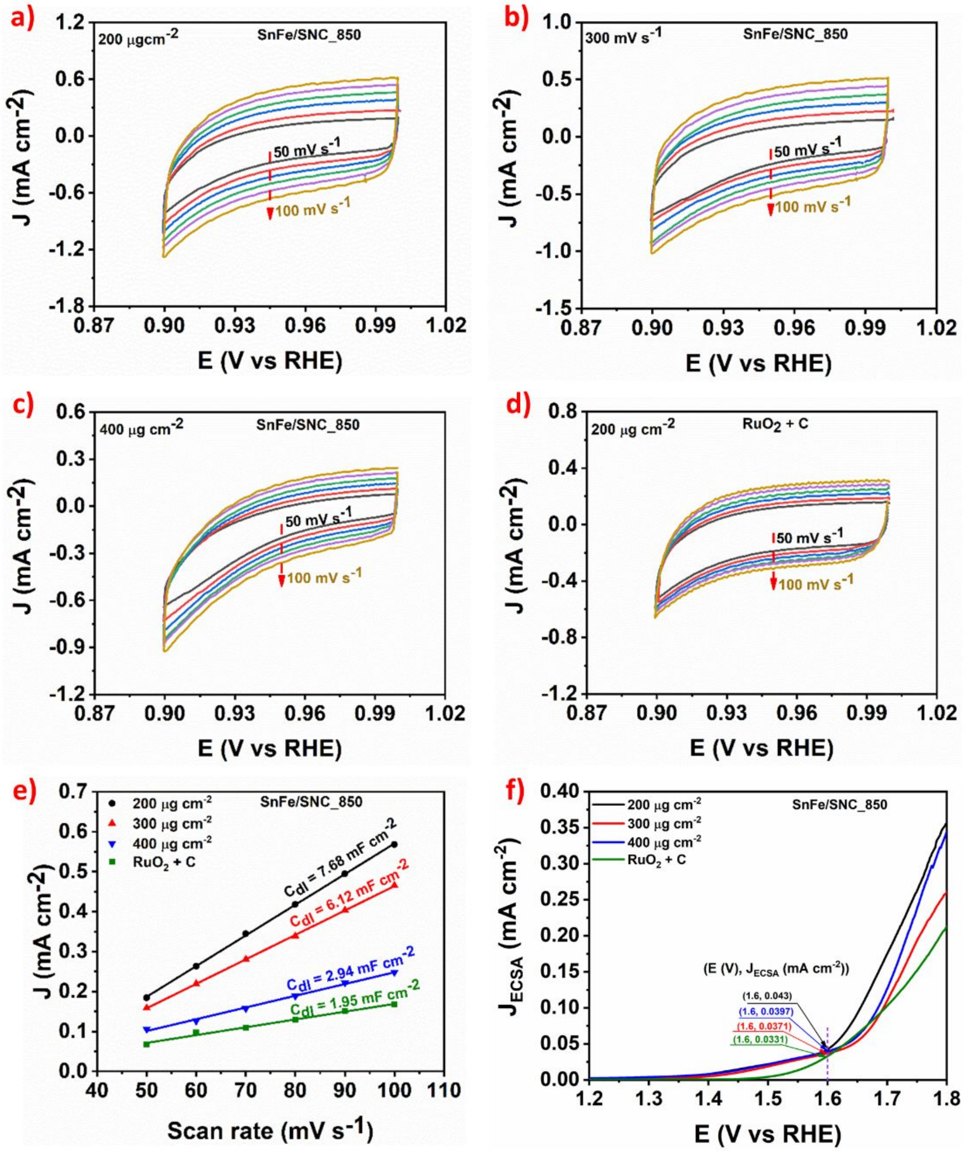

Figure S10. Determination of electrochemically active surface area (ECSA) under OER conditions. Cyclic voltammetry (CV) curves of SnFe/SNC_850 recorded at different catalyst loadings: (a) 200, (b) 300, and (c) 400 μg cm^{-2}, together with (d) commercial RuO_2+C for comparison. (e) Corresponding double-layer capacitance (C_dl) values obtained from the CV measurements. (f) ECSA-normalized OER polarization curves of SnFe/SNC_850 at different catalyst loadings.

The ECSA in the OER condition was calculated from the C_{dl} using equation (1) (see **Figure S5**). Here, the C_s is 40 μF cm^{-2} for the synthesized catalysts and the commercial RuO_2+C in 1 M KOH. The calculated ECSA values are shown in Table S5 below.

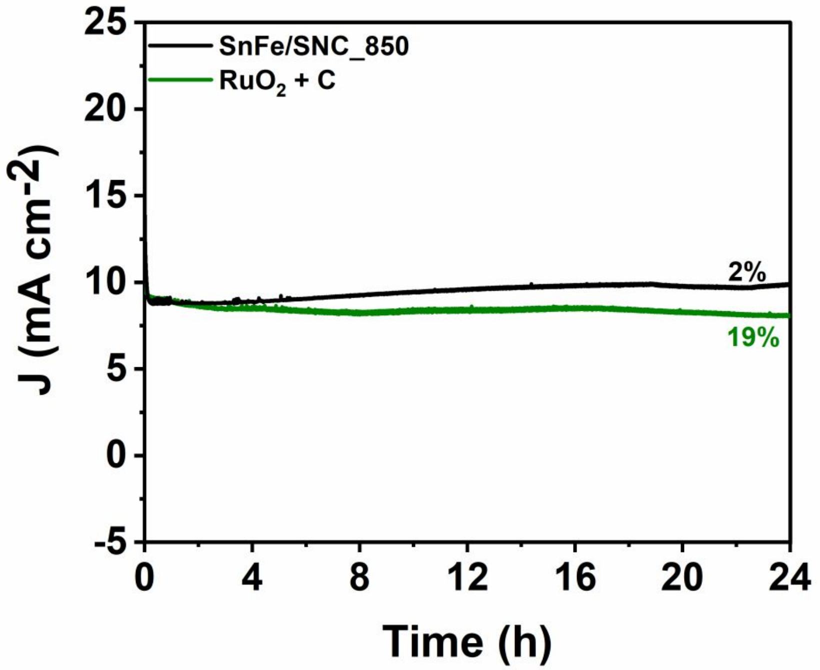

Figure S11. Chronoamperometric curves of SnFe/SNC_850 and commercial RuO_2+C depicting the corresponding OER stabilities.

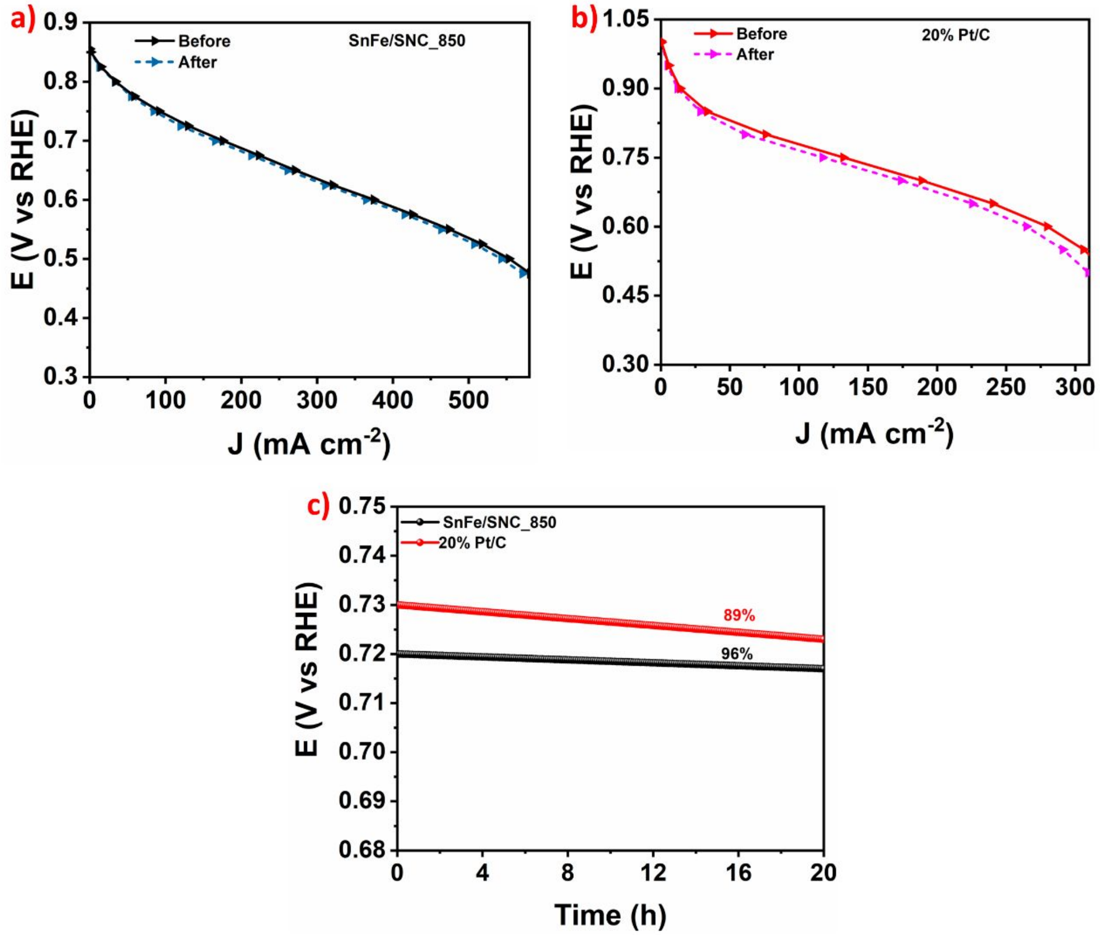

Figure S12. (a) Polarization (E–J) curves of SnFe/SNC_850 before and after 20 h single-cell durability testing at 65 °C under H_2/O_2 (150 kPa(abs)); (b) polarization (E–J) curves of commercial 20 wt% Pt/C cathode measured under identical conditions; and (c) voltage–time (E–t) profiles of SnFe/SNC_850 and 20 wt% Pt/C recorded during constant-current durability operation at 0.3 A cm^{-2}. In the polarization (E-J) curves, solid lines denote the initial performance, whereas dashed lines represent post-durability test results. SnFe/SNC_850 exhibits a lower voltage loss ($\Delta V \approx 30$ mV) and higher current retention (~96 %) than Pt/C ($\Delta V \approx 80$ mV, ~89 % retention), demonstrating excellent structural stability and superior interfacial durability during prolonged AEMFC operation.

Table S1. SEM EDS wt. % and atom % of SnFe/SNC_T (T: 750, 850 , and 950 °C).

	SnFe/SNC_750		SnFe/SNC_850		SnFe/SNC_950	
**Element**	**Wt.%**	**Atomic%**	**Wt.%**	**Atomic%**	**Wt.%**	**Atomic%**
C	33.86	53.57	54.99	75.99	49.41	74.58
N	24.22	32.86	14.39	14.39	11.96	15.48
S	4.19	2.49	1.31	0.74	1.13	0.58
Fe	27.96	9.52	20.40	6.62	17.75	5.25
Sn	9.77	1.56	16.92	2.58	11.73	1.63

Table S2. % Concentration of N and S bond configurations based on high-resolution N1s and S2p XPS spectra of SnFe/SNC_850

N1s		S2p	
**Bond configurations**	**% Concentration**	**Bond configurations**	**% Concenetration**
Pyridinic N	35	M-S_x	17.4
Pyrrolic N/M-N_x	22	C-S	32
Graphitic N	29	C=S	16
Oxidized N	14	C-SO_x	34.6

Table S3. % Concentration of Fe bond configurations (active sites) and oxidation states of Fe based on high-resolution Fe2P XPS spectra of SnFe/SNC_850

Fe2p	
**Bond configurations and oxidation states**	**% Concentration**
Fe-N_x	14.09
Fe-S_x	9.26
F^0	11.56
Fe^{2+}	35.35
Fe^{3+}	21.17
Sat.	8.57

Table S4. ECSA-normalized current densities (J_{ECSA}) for ORR performances of SnFe/SNC_T (T = 750, 850 & 950 °C), and 20% Pt/C

Sample	ECSA	ORR	
		J at 0.85 V vs RHE (mA cm^{-2})	J_{ECSA} at 0.80 V vs RHE (mA cm^{-2})
SnFe/SNC_750	153	-0.95	-0.0062
SnFe/SNC_850	205	-2.91	-0.0142
SnFe/SNC_950	143	-1.12	-0.0078
20% Pt/C	203	-1.85	-0.0091

ECSA normalized J:

- ✓ J_{ECSA} = J/ECSA (2)

Table S5. EIS fitting results (Rs and Rct values) of the synthesized catalysts

Catalysts	R_s (Ω)	R_{ct} (Ω)
SnFe/SNC_750	1.109	15.26
SnFe/SNC_850	1.357	9.314
SnFe/SNC_950	1.124	14.37
20% Pt/C	1.389	11.75

Table S6. Metal elements composition of SnFe/SNC_850 before and after ORR/OER durability test based on ICP-OES analysis

Elements	ORR		OER	
	% composition (before)	% composition (after 30,000 ADT cycles)	% composition (before)	% composition (after 24 h chronoamperometric test)
Fe	7.42	7.38	7.42	7.41
Sn	3.56	3.53	3.56	3.55

Table S7. ECSA-normalized current densities (J_{ECSA}) for OER performances of SnFe/SNC_850 at different loadings and the benchmark RuO_2 +C

Catalyst & loading	ECSA)	OER	
		J at 1.6 V vs RHE (mA cm^{-2})	J_{ECSA} at 1.6 V vs RHE (mA cm^{-2})
SnFe/SNC_850, 200 µg cm^{-2}	192	8.2002	0.043
SnFe/SNC_850, 300 µg cm^{-2}	153	5.676	0.0371
SnFe/SNC_850, 400 µg cm^{-2}	73.5	2.922	0.0397
(RuO_2+C), 200 µg cm^{-2}	48.75	1.613	0.0331

Table S8. Comparison of ORR activity of SnFe/SNC_850 and its fuel cell performance based on power density with previously reported dual metallic electrocatalysts.

Catalysts	$E_{1/2}$ (V) of ORR curve	Power density of AEMFC (mW cm^{-2})	References
**SnFe/SNC_850**	**0.86**	**277**	**This work**
**FeCo-1/NSC**	0.82	162.74	(3) Chang, S., Zhang, H., and Zhang, Z. (2021) FeCo alloy/N, S dual-doped carbon composite as a high-performance bifunctional catalyst in an advanced rechargeable zinc-air battery. Journal of Energy Chemistry **56**, 64-71.
**NiCo2-CPO-27/PCN-HT**	0.82	224.4	(15) Pradesar, Y., Yusuf, A., Kabtamu, D.M., et al. (2023) Nickel–Cobalt Metal–Organic Framework CPO-27 and g-C3N4 for Oxygen Reduction Reaction in Alkaline-Exchange-Membrane Fuel Cell. ACS Applied Energy Materials 6, 7847-7856.

**CoFe/S-N-C**	0.855	130	(5) Li, G., Tang, Y., Fu, T., et al. (2022) S, N co-doped carbon nanotubes coupled with CoFe nanoparticles as an efficient bifunctional ORR/OER electrocatalyst for rechargeable Zn-air batteries. Chem. Eng. J. **429**, 132174.
**FeS_2-CoS_2/NCFs**	0.81	257	(68) Shi, X., He, B., Zhao, L., et al. (2021) FeS_2–CoS_2 incorporated into nitrogen-doped carbon nanofibers to boost oxygen electrocatalysis for durable rechargeable Zn-air batteries. J. Power Sources **482**, 228955.
**Se–CoSe2/CNFs**	0.80	149.4	(23) Cui, W., Xu, S., Bai, J., et al. (2024) ZIF-67-derived Se-doped CoSe 2 grown on carbon nanofibers as oxygen electrocatalysts for rechargeable Zn–air batteries. New Journal of Chemistry **48**, 4310-4319.
**FeNi-NC@MWCNT**	0.90	218.3	(8) Chen, Z., Cheng, W., Cao, K., et al. (2025) A Bifunctional Iron-Nickel Oxygen Reduction/Oxygen Evolution Catalyst for High-Performance Rechargeable Zinc-Air Batteries. Small **21**, 2409161.
**Mo/Fe/Co@NC**	0.85	150	(9) Li, S., Zhou, Y., Xu, C., et al. (2024) ZIFs-Derived Hollow Nanostructures via a Strong/Weak Coetching Strategy for Long-Life Rechargeable Zn-Air Batteries. Small **20**, 2309932.
**RuPt DSAs-NC**	0.86	171	(10) Hu, X., Wu, Z., and Xu, C. (2024) Precise construction of RuPt dual single-atomic sites to optimize oxygen

			electrocatalytic behaviors for high-performance Zn-air batteries. Journal of Energy Chemistry **97**, 520-528.
**CoMn/NC**	0.89	176	(11) Dey, G., Jana, R., Saifi, S., et al. (2023) Dual single-atomic Co–Mn sites in metal–organic-framework-derived N-doped nanoporous carbon for electrochemical oxygen reduction. ACS Nano **17**, 19155-19167.
**CoFe/Se@CN**	0.87	160	(16) Dai, L., Feng, C., Luo, Y., et al. (2024) CoFe Alloys Dispersed on Se, N Co-Doped Graphitic Carbon as Efficient Bifunctional Catalysts for Zn-Air Batteries. Chemistry–A European Journal **30**, e202303173.
**CoSn@NC**	0.813	---	(20) Dong, C., Zhang, X., Zhang, S., et al. (2023) Manipulating oxygenate adsorption on N-doped carbon by coupling with CoSn alloy for bifunctional oxygen electrocatalyst. Green Energy & Environment **8**, 1417-1428.
**SnSb-NC**	0.87	198.5	(21) Li, S., Han, J., Guo, Y., et al. (2023) A honeycomb carbon substrate anchored with Sn and Sb bimetallic atoms boosts oxygen-reduction electrocatalysis. Inorg. Chem. Front. **10**, 3568-3576.
**CoNi(1:1)-TB-800N2**	0.888	154.8	(45) He, X., Yin, F., Li, G., et al. (2020) CoNi alloys with slight oxidation@ N, O Co-doped carbon: enhanced collective contributions of cores and shells to multifunctional electrocatalytic activity

			and Zn–air batteries. Journal of Materials Chemistry A **8**, 25805-25823.
